# Pharmacological interventions for remifentanil-induced hyperalgesia: A systematic review and network meta-analysis of preclinical trials

**DOI:** 10.1371/journal.pone.0313749

**Published:** 2024-12-05

**Authors:** Mia E. Koponen, Emily Naray, Tim G. Hales, Patrice Forget

**Affiliations:** 1 MSc Clinical Pharmacology, School of Medicine, Medical Sciences and Nutrition, University of Aberdeen, Aberdeen, United Kingdom; 2 MSci Biomedical Sciences, School of Medicine, Medical Sciences and Nutrition, University of Aberdeen, Aberdeen, United Kingdom; 3 Division of Systems Medicine, School of Medicine, Institute of Academic Anaesthesia, Ninewells Hospital, University of Dundee, Dundee, United Kingdom; 4 Institute of Applied Health Sciences, Epidemiology Group, School of Medicine, Medical Sciences and Nutrition, University of Aberdeen, Aberdeen, United Kingdom; 5 Department of Anaesthesia, National Health Service (NHS) Grampian, Aberdeen, United Kingdom; 6 Emergency and Pain Medicine Division, IMAGINE UR UM 103, Montpellier University, Anesthesia Critical Care, Nîmes University Hospital, Nîmes, France; 7 Pain and Opioids after Surgery (PANDOS) European Society of Anaesthesia (ID ESAIC_RG_PAND) Research Group, Brussels, Belgium; Sapienza University of Rome: Universita degli Studi di Roma La Sapienza, ITALY

## Abstract

**Background:**

To improve perioperative pain management, several interventions have been suggested for the prevention of increased pain sensitivity caused by opioids (called opioid-induced hyperalgesia). It is currently unclear which intervention is the most effective or appropriate in preventing opioid-induced hyperalgesia. Remifentanil is the most investigated opioid causing opioid-induced hyperalgesia. Thus, to guide future research, we conducted a systematic review and a network meta-analysis of preclinical trials investigating pharmacological interventions for remifentanil-induced hyperalgesia.

**Methods:**

To identify relevant articles, electronic database searches were conducted in Embase, PubMed, Web of Science, and Google Scholar. Study characteristics were extracted, and the risk of bias was evaluated. Studies were included in the network meta-analysis if they shared similar characteristics with at least one other study. The interventions were ranked based on P-scores.

**Results:**

Overall, the 62 eligible trials tested 86 individual interventions and 6 combination interventions. Thirty-five studies eligible in the network meta-analysis formed five groups which were further divided into subgroups based on the quantitative sensory tests used. The best-ranked interventions within the subgroups were Anxa1_2-26_, MRS2179, salicylaldehyde isonicotinoyl hydrazone (SIH), ANA-12, TDZD-8, ketamine, dexmedetomidine, JWH015, and the combination of KN93 and ketamine.

**Discussion:**

The current literature is too heterogeneous to produce a clear answer on which intervention is the most effective in preventing remifentanil-induced hyperalgesia. Future research in this field should prioritise finding the most effective intervention over testing the efficacy of new options. The results of our work can be used in planning which comparisons should be included in new trials.

## Introduction

Opioids are widely considered to be the best choice of analgesics for moderate and severe pain and as adjuncts to general anaesthetics [1]. Particularly, remifentanil (a potent short-acting opioid) is commonly used during surgery [2]. Yet, opioids have many potential serious side-effects such as respiratory depression and impaired gastro-intestinal function, and their use can lead to addiction [3]. Opioid administration may also be followed by an increase in pain sensitivity—a condition called opioid-induced hyperalgesia (OIH) [4,5]. The various cellular and molecular mechanisms that contribute to the development of OIH have been reviewed elsewhere [6]. The clinical relevance of OIH has been debated despite it being a well-demonstrated phenomenon in preclinical trials [7,8]. Interestingly, reviews of randomised controlled trials suggest that remifentanil does not induce hyperalgesia to a clinically significant degree in healthy volunteers [9,10] whilst a clinically significant effect is observed in postsurgical patients [11]. Clinical trials investigating remifentanil-induced hyperalgesia (RIH) show that it is associated with more intense acute postsurgical pain and a higher likelihood of developing chronic postsurgical pain. A systematic review and meta-analysis of randomised controlled trials comparing different intra-operative opioid doses in 1,494 adults undergoing surgery concluded that high intra-operative doses of remifentanil were associated with more intense pain during the 24 hours after surgery [11]. In addition, a study that evaluated 28 adults undergoing surgery with high or low dose remifentanil found that the incidence of chronic postsurgical pain during the 6–13 months follow-up was higher in the high remifentanil dose group [12]. Moreover, many clinicians conclude that it is becoming clearer that OIH is a significant clinical challenge [5,7,13]. Raffa and Pergolizzi (2013) [13] argue that OIH is one of the most understudied, important aspects of opioid research. Despite these conclusions, there are no guidelines for preventing OIH.

The current literature provides a wide variety of pharmacological and non-pharmacological interventions for preventing OIH including dexmedetomidine, ketamine, magnesium sulfate, exercise, polyamine-deficient diet, and electroacupuncture therapy [14–17]. Until very recently, there has been no literature evaluating which intervention is the most effective or appropriate. Xie et al. (2023) [18] published the first-ever network meta-analysis (NMA) to rank the efficacy of the pharmacological interventions for OIH. They reviewed 33 randomised controlled clinical trials which in total evaluated 19 interventions. The results showed that amantadine was the most effective in controlling acute postsurgical pain. It should be noted that their NMA included trials which used different types of surgeries, different opioids, and different opioid infusion rates and durations. Previous research suggests that the mechanism underlying OIH caused by remifentanil is different from that caused by morphine or fentanyl [19], meaning that the efficacy of interventions should be evaluated separately for different opioids. Similarly, it has been demonstrated that different opioid infusion rates and durations result in significantly different levels of OIH [20]. Different levels of OIH and different surgery types would result in considerably different levels of pain between the participants in different trials. Since pain relief does not occur linearly across different levels of pain intensity [21], this heterogeneity between the trials undermines the ranking of the interventions by Xie et al. (2023) [18]. Ultimately, this leaves clinicians without any guidance for selecting the best intervention for OIH. While randomised controlled trials on OIH are currently too heterogeneous for ranking interventions, our scoping review suggests that preclinical trials may provide adequately homogenous groups for NMAs [17]. Thus, to rank the efficacy of pharmacological interventions for RIH, we conducted a systematic review and a network meta-analysis of the relevant preclinical trials.

## Methods

### Protocol and registration

The protocol of this systematic review and network meta-analysis was registered on the PROSPERO international prospective register of systematic reviews (Registration ID: CRD42023432273). This report follows the Preferred Reporting Items for Systematic Reviews and Meta-analysis extension for Network Meta-Analyses (PRISMA-NMA) checklist [22].

### Search strategy

Embase, PubMed, Web of Science, and Google Scholar were searched in June 2023. The search strategy combined keywords or medical subject headings for remifentanil-induced hyperalgesia, preclinical trials, and interventions. The lists of search terms for preclinical trials were obtained from van der Mierden et al. (2022) [23]. The detailed search strategy is available in S1 Table. The retrieved records were screened independently by two reviewers (MK & EN) and discrepancies were resolved by discussion. Relevant reviews and reference lists of eligible articles were also screened.

### Eligibility criteria

For a trial to be eligible in the review, it had to (1) investigate a pharmacological intervention for remifentanil-induced hyperalgesia, (2) use *in vivo* animal model without pre-existing disease or genetic modification, (3) measure hyperalgesia using a quantitative sensory test [24] (4) have a comparison group: placebo, vehicle, no-intervention, or another pharmacological intervention, (5) be a full original research paper rather than a review or abstract, and (6) be written in English. Pharmacological interventions with all timings, frequencies, dosages, and administration methods were included. No publication time period restrictions were set.

### Data extraction

Study characteristics data extraction was conducted by one reviewer (MK) and checked for accuracy by another reviewer (EN). Discrepancies were resolved by discussion. The eligible studies were categorised into three groups: studies with plantar incision (a standard postoperative pain model [25]), studies without plantar incision, and studies with and without plantar incision. The extracted information included general information (the first author, year of publication, title, and digital object identifier), remifentanil characteristics (route of administration, infusion rate, infusion duration), anaesthesia drug, animal model characteristics (species, strain, sex, age, weight), intervention characteristics (drug name, mechanism of action, route of administration, dose, time of administration compared to remifentanil administration), type and size of study groups, total number of study groups, quantitative sensory test (QST) characteristics (test type, times of measurement, body part used in measurements [e.g. hind paw/tail, ipsilateral/contralateral hind paw compared to plantar incision]), whether QST data was presented in text/tables/figures, and relevant figure number. Primary outcome data (QST values) was only extracted from studies that were eligible in the network meta-analysis (NMA). When primary outcome data was only provided in a figure and not in writing or a table, the author of correspondence was emailed twice to request the data. If no data was provided, one reviewer used Plot Digitizer to obtain the mean and standard deviation (SD) of relevant data points. When standard error of the mean (SEM) was given instead of SD, the formula SD = SEM*√n was used to calculate the SD.

### Risk of bias assessment

Two reviewers (MK & EN) assessed the risk of bias in each study independently using the Systematic Review Centre for Laboratory Animal Experimentation (SYRCLE) risk of bias tool [26]. The tool includes ten criteria: (1) sequence generation, (2) baseline characteristics, (3) allocation concealment, (4) random housing, (5) blinding against performance bias, (6) random outcome assessment, (7) blinding against detection bias, (8) incomplete outcome data, (9) selective outcome reporting, and (10) other sources of biases. Each aspect was marked as “low risk”, “high risk” or “unclear risk”. Discrepancies in the judgements by the two reviewers were resolved by discussion.

### Statistical analysis

For a study to be eligible in the NMA, it had to share similar plantar incision status, remifentanil characteristics, animal model, and QST type with at least one other study. The most common time point of QST measurements were chosen for the analysis for each group of similar studies. Studies that did not share the most common time point with other studies of the group were excluded. In addition, studies that reported data only as median and interquartile range (IQR), reported data only as a percentage change to baseline, or did not report SD or SEM were excluded. A NMA was conducted for each group of similar studies. First, network plots were created using Confidence in Network Meta-Analysis (CINeMA) web application [27]. Second, the mean differences were compared in a random effects model and league tables with 95% confidence intervals (CIs) were obtained. Forest plots were created to display the mean difference (MD) and 95% CI of each intervention compared to the no-intervention condition. Finally, the interventions were ranked based on P-scores which take in account the mean differences, confidence intervals, and sample sizes [28]. These were obtained using NMA studio: An interactive tool for network meta-analyses [29].

## Results

### Study selection

**Fig 1:**
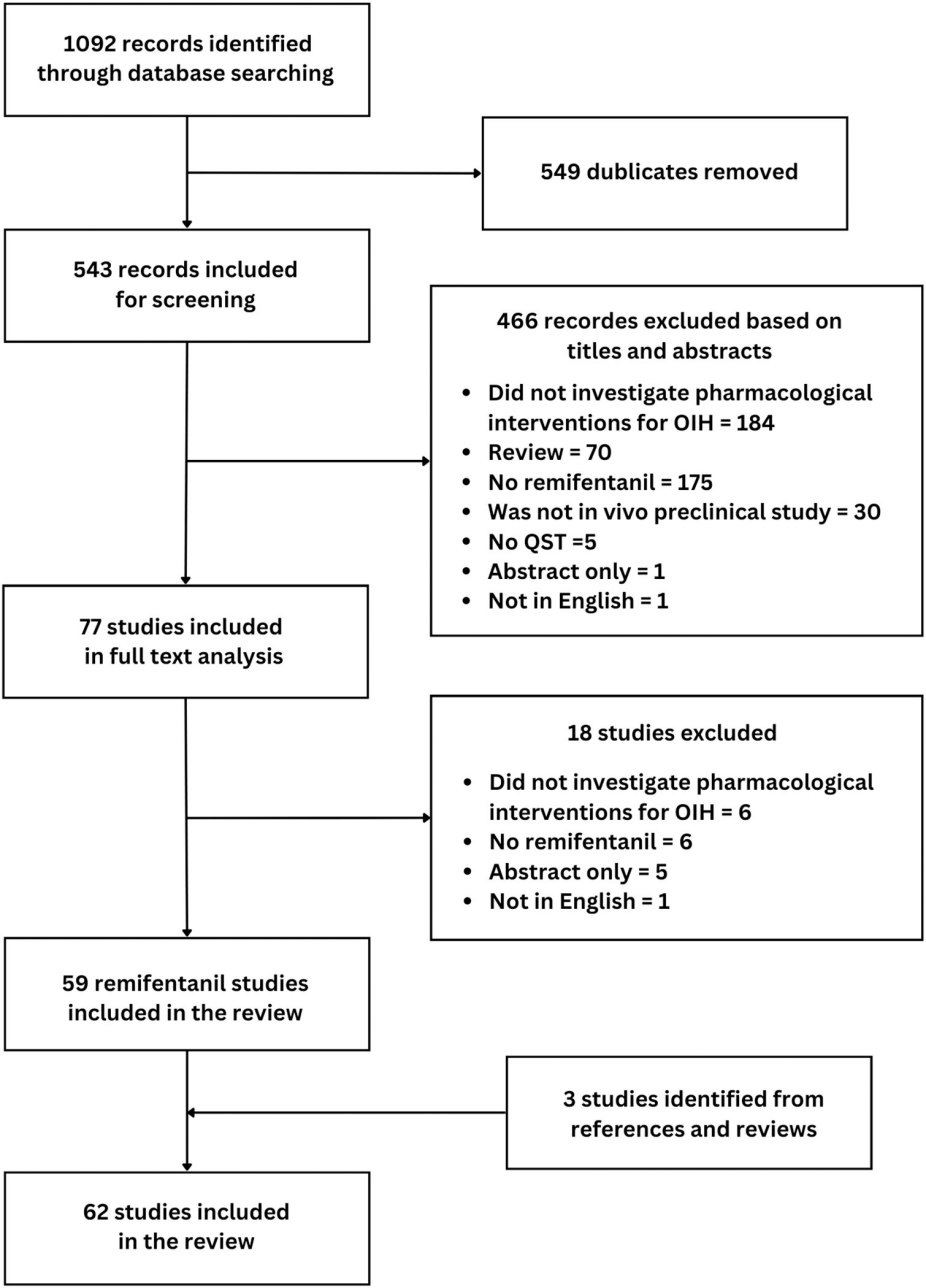
Flowchart of the study selection process. Our search identified 1092 records. After deleting duplicates, 543 titles and abstracts were screened. Sixty-two articles were included in the review.

Fig 1 summarises the study selection process. The search in Embase, PubMed, Web of Science, and Google Scholar resulted in 1092 potentially relevant records. After duplicate deletion, 543 records were included for screening. Title and abstract screening excluded 466 records, and full text screening excluded 18 records. Sixty-two articles were included in the review after 3 additional articles were identified from screening relevant reviews and reference lists of eligible articles.

### Study characteristics

Study characteristics extracted for each study are available in S2 Table. Out of the 62 included studies, 33 conducted plantar incision, 23 did not conduct plantar incision, and 6 studies had experiments with and without plantar incision. Generally, remifentanil was administered as an intravenous (IV) or subcutaneous (SC) infusion for 30–120 minutes. Anaesthesia was most commonly induced with sevoflurane. Most of the studies (51/62) were conducted with adult male Sprague-Dawley rats, while the rest used Wistar rats, C57BL/6J mice, ICR mice, or CD1 mice. Female rodents were only included in two studies [30,31]. The majority of the studies (54/61) conducted both mechanical and thermal QSTs on the hind paw of the rodent. These were usually conducted with electronic von Frey (EVF) device or von Frey monofilaments (VFM) and hotplate or radiant heat test. The most common combination of QST time points was 2 h, 6 h, 24 h, and 48 h.

### Risk of bias assessment

**Fig 2:**
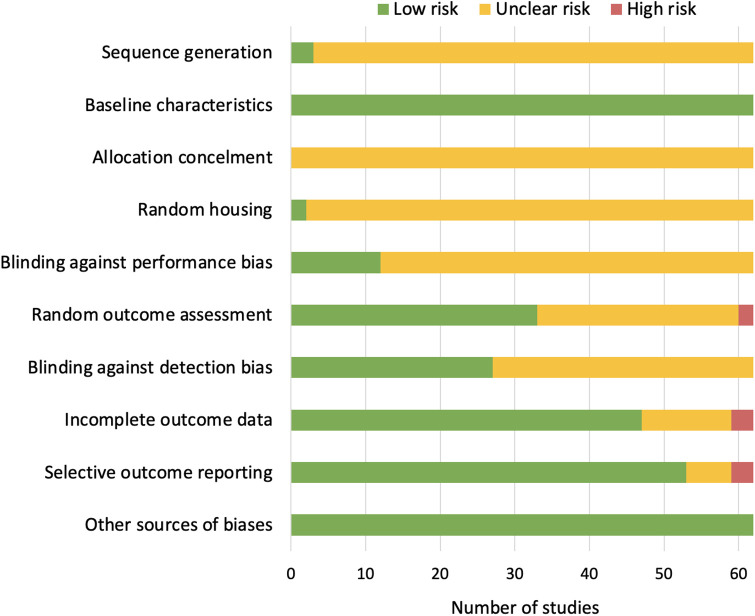
Overall risk of bias. The reviewed studies mostly have low risk of bias or unclear risk of bias. Overall, sequence generation, allocation concealment, random housing, and blinding against performance bias were rarely reported. Only three studies had items judged to be high risk of bias.

Fig 2 shows the overall SYRCLE risk of bias assessment. Risk of bias of individual studies is available in S3 Table. The reviewed studies mostly have low risk of bias or unclear risk of bias. Overall, sequence generation, allocation concealment, random housing, and blinding against performance bias were rarely reported. The lack of information about these domains is likely to reflect the limitations of preclinical research in general rather than potentially increased risk of bias in the studies included in our review. Only three studies had items judged to be high risk of bias. These concerned incomplete outcome data, selective outcome reporting, and random outcome assessment.

### Interventions

A total of 86 individual interventions and six combination interventions were investigated. The individual interventions are listed in Table 1 in alphabetical order. The combination interventions were: hydrogen-rich saline and Ro 25–6981 [32], hydrogen-rich saline and MK801 [32], PHA-543613 and PNU-120596 [33], ketamine and KN93 [34], CLP257 and muscimol [35], and artesunate and MPEP [36].

**Table 1:** Pharmacological interventions for RIH.

Intervention	Studied by
A438079	Yuan et al. (2022) [37]
ACET	Wang et al. (2023) [38]
Ac-YVAD-CMK	Yuan et al. (2022) [37] Li et al. (2023) [39]
AgomiR-134	Wang et al. (2023) [38]
ANA-12	Fu et al. (2021) [30]
Anxa1_2-26_	Li et al. (2019) [40]
AMD3100	Li et al. (2019) [40]
Amitriptyline	Aguado et al. (2015) [41]
Artesunate	Zhang et al. (2022) [36]
Betulinic acid	Lv et al. (2018) [42]
CAY10444	Li et al. (2023) [39]
Chelerythrine	Li et al. (2017) [43]
CCL1 neutralising antibody	Zhang et al. (2015)a [44]
CCL3 neutralising antibody	Li et al. (2016) [45]
CCL7 neutralising antibody	Qiang and Yu (2019) [46]
CCL21 neutralising antibody	Wang et al. (2020)a [47]
CLP257	Gao et al. (2020) [48]
CX3CR1 neutralising antibody	Gong et al. (2016) [49]
CXCL13 neutralising antibody	Zhu et al. (2017) [50]
CYM-5442	Li et al. (2023) [39]
CYM-5478	Li et al. (2023) [39]
EphB1-Fc	Xia et al. (2014) [51]
EphB2-Fc	Peng et al. (2019) [52]
Dezocine	Zhou et al. (2020) [53]
Deferoxamine	Shu et al. (2021) [54]
Dexmedetomidine	Zheng et al. (2012) [55] Yuan et al. (2017) [56]
Dynamin-related protein 1 antisense oligodeoxynucleotides	Zhou et al. (2023) [57]
FR167653	Horii et al. (2020) [58]
FTY720	Li et al. (2023) [39]
Fz-8/Fc	Gong et al. (2016) [49]
Hevin-shRNA	Wang et al. (2020)b [59]
HOE-140	Horii et al. (2020) [58]
Hydrogen rich saline	Zhang et al. (2014)b [60] Zhang et al. (2014)a [44] Shu et al. (2015) [61]
IL-1ra	Yuan et al. (2022) [37]
IL-17 antiserum	Zhu et al. (2017) [50]
IL-18BP	Qiang and Yu (2019) [46]
IWP-2	Gao et al. (2020) [48]
JWH015	Sun et al. (2014) [62]
Kalirin-7 shRNA	Zhang et al. (2018) [63]
Ketamine	Abreu et al. (2015) [64] Gu et al. (2009) [65] Qi et al. (2020) [34] Sun et al. (2016) [66]
KN93	Li et al. (2017) [43] Jiang et al. (2015) [67] Qi et al. (2020) [34]
LHVS	Ye et al. (2017) [68]
LiCl	Li et al. (2014) [69]
Lidocaine	Cui et al. (2015) [70] Wang et al. (2018) [71] Cui et al. (2009) [72]
LT1002	Li et al. (2023) [39]
N-acetyl-cysteine	Liu et al. (2017) [73]
NASPM	Zhang et al. (2018) [63]
Naloxone / (+)-naloxone	Aguado et al. (2013) [74] Yuan et al. (2022) [37] Horii et al. (2020) [58]
Naltrindole	Liu et al. (2018) [75] Wang et al. (2015) [76]
NBI-74330	Wang et al. (2020)a [59]
NMDA	Mert et al. (2014) [31]
NPC-15437	Zhao et al. (2017) [77]
NS398	Peng et al. (2019) [52]
Magnesium	Sun et al. (2016) [66] Sun et al. (2017) [78]
Maraviroc	Li et al. (2016) [45]
Maropitant	Aguado et al. (2015) [41]
MCC950	Li et al. (2023) [39]
Methylnaltrexone	Horii et al. (2020) [58]
Minocycline	Aguado et al. (2015) [41] Ye et al. (2017) [68]
MK-801	Mert et al. (2014) [31] Zhang et al. (2014)b [79] Xia et al. (2014) [51] Su et al. (2021) [80]
MPEP	Zhang et al. (2022) [36]
MRS2179	Su et al. (2021) [80]
Muscimol	Gao et al. (2022) [35]
PBN	Ye et al. (2017) [68] Li et al. (2023) [39] Ye et al. (2016) [81]
PD98059	Li et al. (2017) [43]
Philanthotoxin-7,4	Li et al. (2021) [82]
PHA-543613	Zhang et al. (2015)b [83]
PNU-120596	Zhang et al. (2015)b [83] Gu et al. (2017) [84]
Ro25-6981	Zhao et al. (2017) [77] Jiang et al. (2013) [85] Zhang et al. (2014)a [32] Gao et al. (2020) [48]
Roscovitine	Liu et al. (2014) [86]
Ru360	Lu et al. (2018) [87]
SB203580	Deng et al. (2016) [88]
SB225002	Yang et al. (2016) [89]
SC58125	Peng et al. (2019) [52]
SEW2871	Li et al. (2023) [39]
SHPE	Cabañero et al. (2009) [20]
SIH	Shu et al. (2015) [61]
SK-1	Li et al. (2023) [39]
TASP0277308	Li et al. (2023) [39]
TDZD-8	Li et al. (2013) [90] Zhang et al. (2014)b [60] Yuan et al. (2013) [91] Li et al. (2014) [69]
TNP-ATP	Fu et al. (2021) [30]
TMEM16C overexpression	Li et al. (2021) [82]
TrkB/Fc	Gu et al. (2017) [84]
U0126	Ishida et al. (2012) [92]
VEID-fmk	Wang et al. (2020)a [47]
Zeta inhibitory peptide	Zhao et al. (2017) [77] Zhang et al. (2018) [63]

Only half of the studies (32/62) investigated more than one intervention. These studies and the interventions they examined are shown in S3 Table. Only three studies investigated more than three interventions. Yuan et al. (2022) [37] and Horii et al. (2020) [58] compared four interventions and Li et al. (2023) [39] compared 11 interventions. Similarly, most of the interventions (46/62) were only tested in one study. S4 Table shows the interventions that were investigated more than once and the studies where they were investigated. The most studied interventions were ketamine, Ro25-6981, TDZD-8, and MK-801—all of which were examined in four studies.

### Studies included in the statistical analysis

The eligible studies (35/62) were categorised into 5 groups based on plantar incision status, remifentanil characteristics, and animal model. These groups were divided into subgroups based on mechanical and thermal QST measurements. Table 2 shows the characteristics of each group of studies. Most of the studies excluded from the statistical analysis (22/27) did not share similar remifentanil characteristics with any other study. The other studies were excluded because they either did not have a QST measurement at 24 hours, reported data only as median and interquartile range, reported data only as a percentage change to baseline, or did not report SD or SEM.

**Table 2:** Characteristics of the groups of studies included in the statistical analysis.

	Plantar incision	Remifentanil characteristics	Animal model	QST	Number of studies
Group 1A	No	IV infusion of 1.0 μg/kg/min for 60 min	Male Sprague-Dawley rat	Von Frey	13
Group 1B	No	IV infusion of 1.0 μg/kg/min for 60 min	Male Sprague-Dawley rat	Hot plate	13
Group 2A	Yes	IV infusion of 1.0 μg/kg/min for 60 min	Male Sprague-Dawley rat	Von Frey	11
Group 2B	Yes	IV infusion of 1.0 μg/kg/min for 60 min	Male Sprague-Dawley rat	Hot plate	9
Group 2C	Yes	IV infusion of 1.0 μg/kg/min for 60 min	Male Sprague-Dawley rat	Radiant heat test	2
Group 3A	Yes	IV infusion of 1.2 μg/kg/min for 60 min	Male Sprague-Dawley rat	Von Frey	4
Group 3B	Yes	IV infusion of 1.2 μg/kg/min for 60 min	Male Sprague-Dawley rat	Radiant heat test	3
Group 4A	Yes	SC infusion of 0.04 mg/kg for 30 min	Male Sprague-Dawley rat	Von Frey	7
Group 4B	Yes	SC infusion of 0.04 mg/kg for 30 min	Male Sprague-Dawley rat	Radiant heat test	7
Group 5A	Yes	SC infusion of 0.04 mg/kg for 30 min	Male ICR mouse	Von Frey	2
Group 5B	Yes	SC infusion of 0.04 mg/kg for 30 min	Male ICR mouse	Hot plate	2

Five groups of studies are divided into subgroups based on QST tests.

### Network plots

Network plots for the 5 groups analysed are shown in S1–S8 Figs. Different doses of the same interventions are categorised into different nodes. Only one network plot per group is provided when the mechanical and thermal groups were identical (Group 1, Group 4, and Group 5). For Group 2, three network plots are provided since two thermal subgroups were analysed. In most networks, the investigated interventions have been compared with the no-intervention control group, but otherwise direct comparisons are limited.

## Forest plots

**Fig 3:**
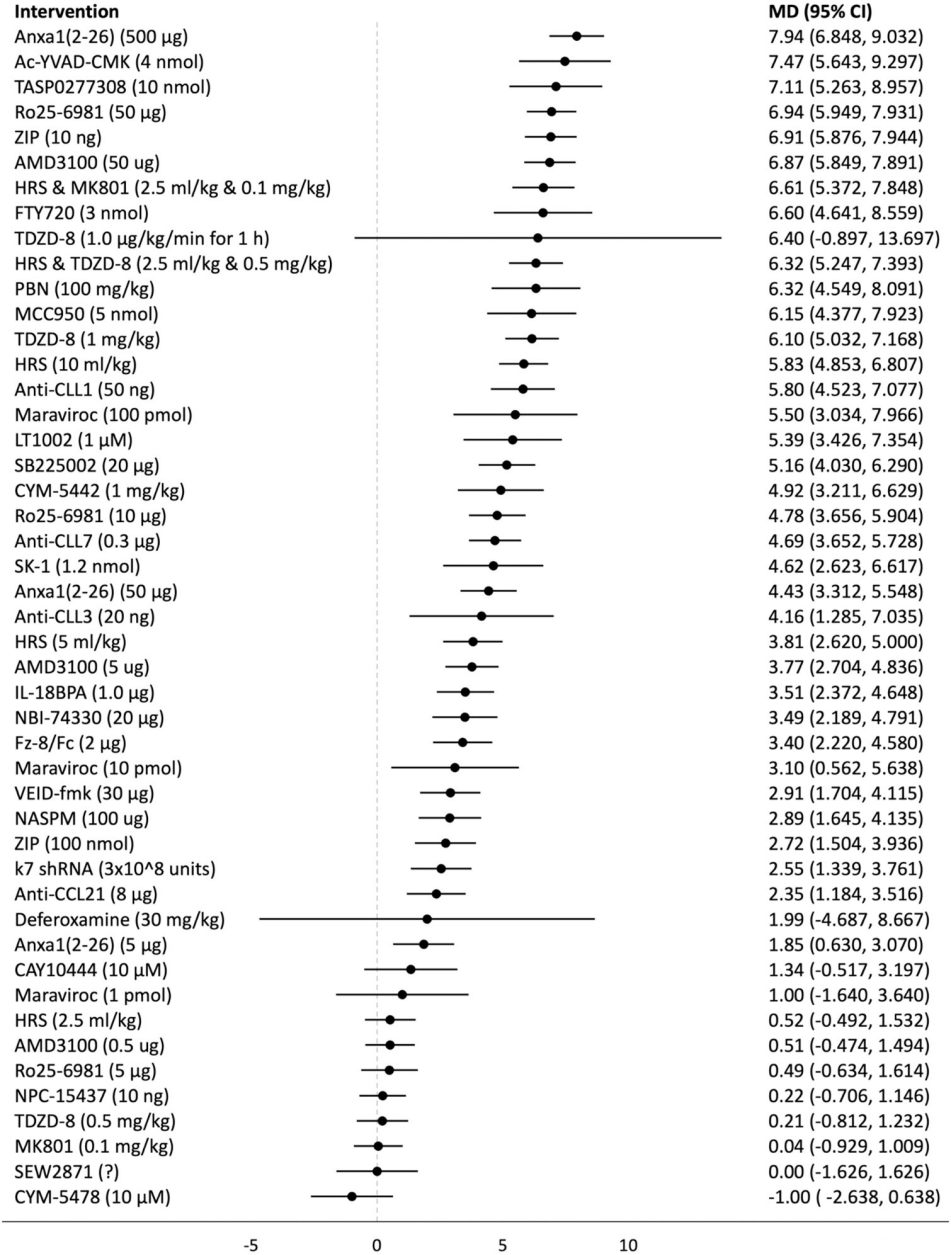
Forest plot for Group 1A. Most of the interventions provided a significant increase in PWT compared to the no-treatment condition. Anxa1_2-26_ (500 μg) had the largest MD.

**Fig 4:**
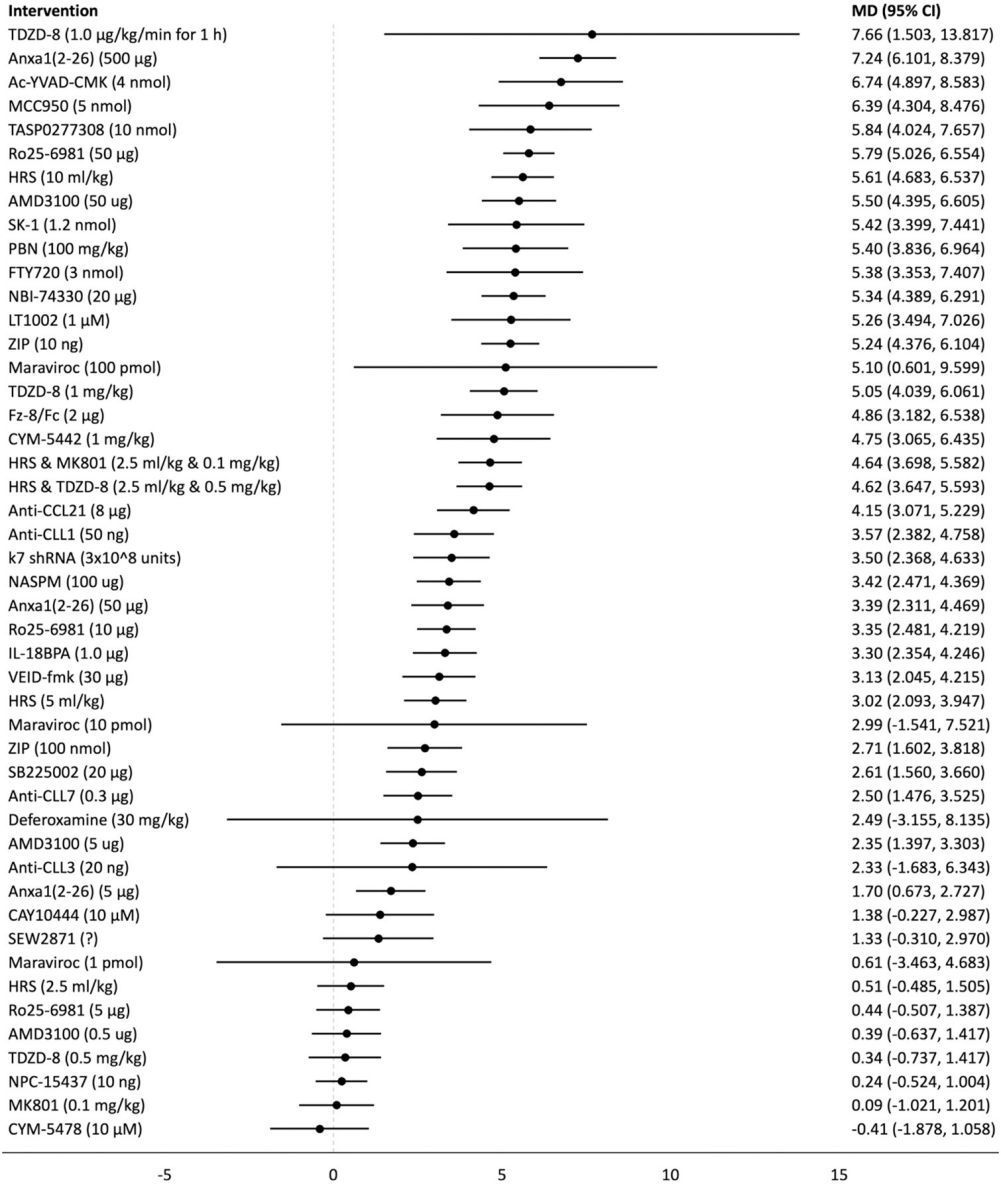
Forest plot for Group 1B. Most of the interventions provided a significant increase in PWT compared to the no-treatment condition. TDZD-8 (1.0 μg/kg/min for 1 h) had the largest MD but it has a large 95% CI. Anxa1_2-26_ (500 μg) had the second largest MD.

**Fig 5:**
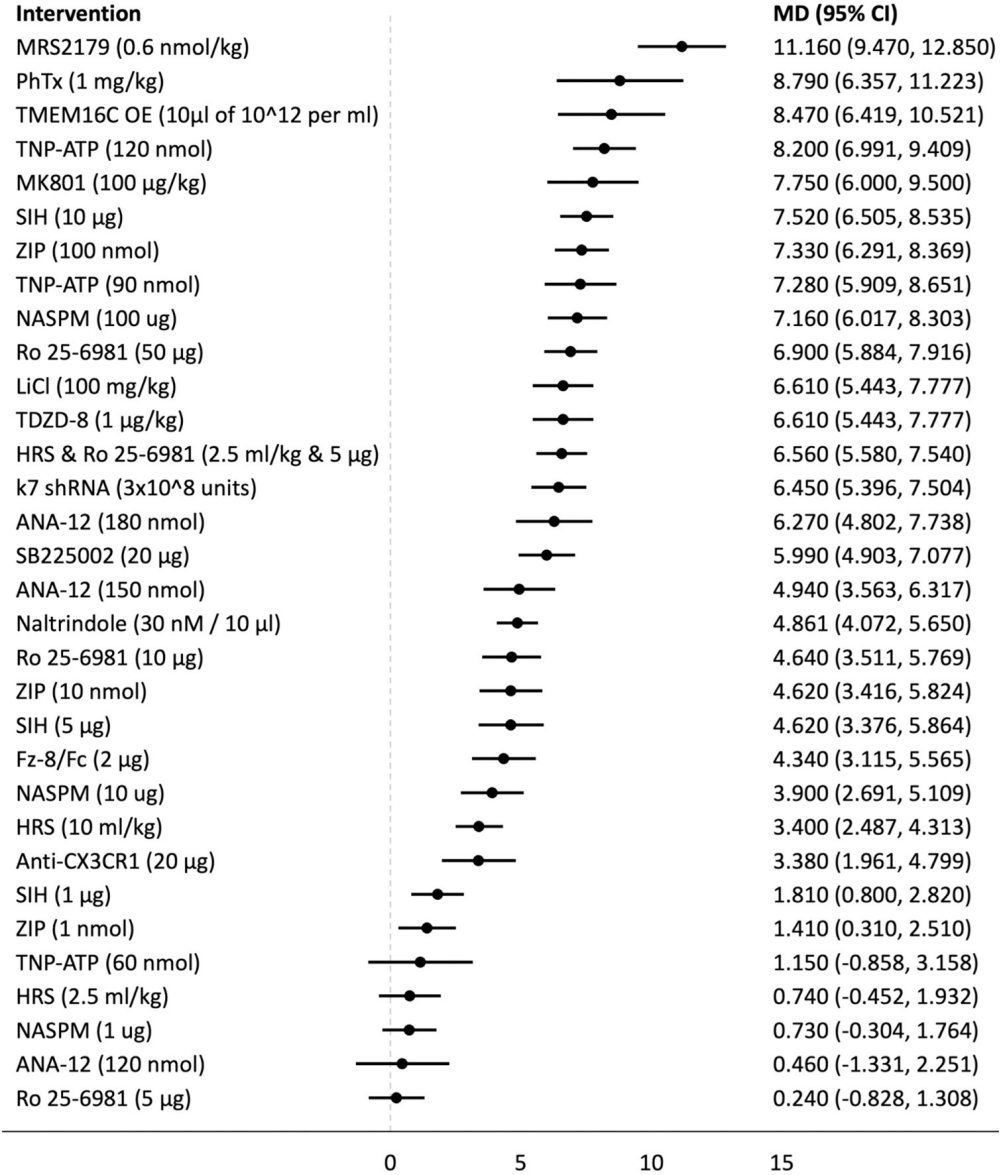
Forest plot for Group 2A. Most of the interventions provided a significant increase in PWT compared to the no-treatment condition. MRS2179 (0.6 nmol/kg) had the largest mean difference and it stands out from the rest of the interventions.

**Fig 6:**
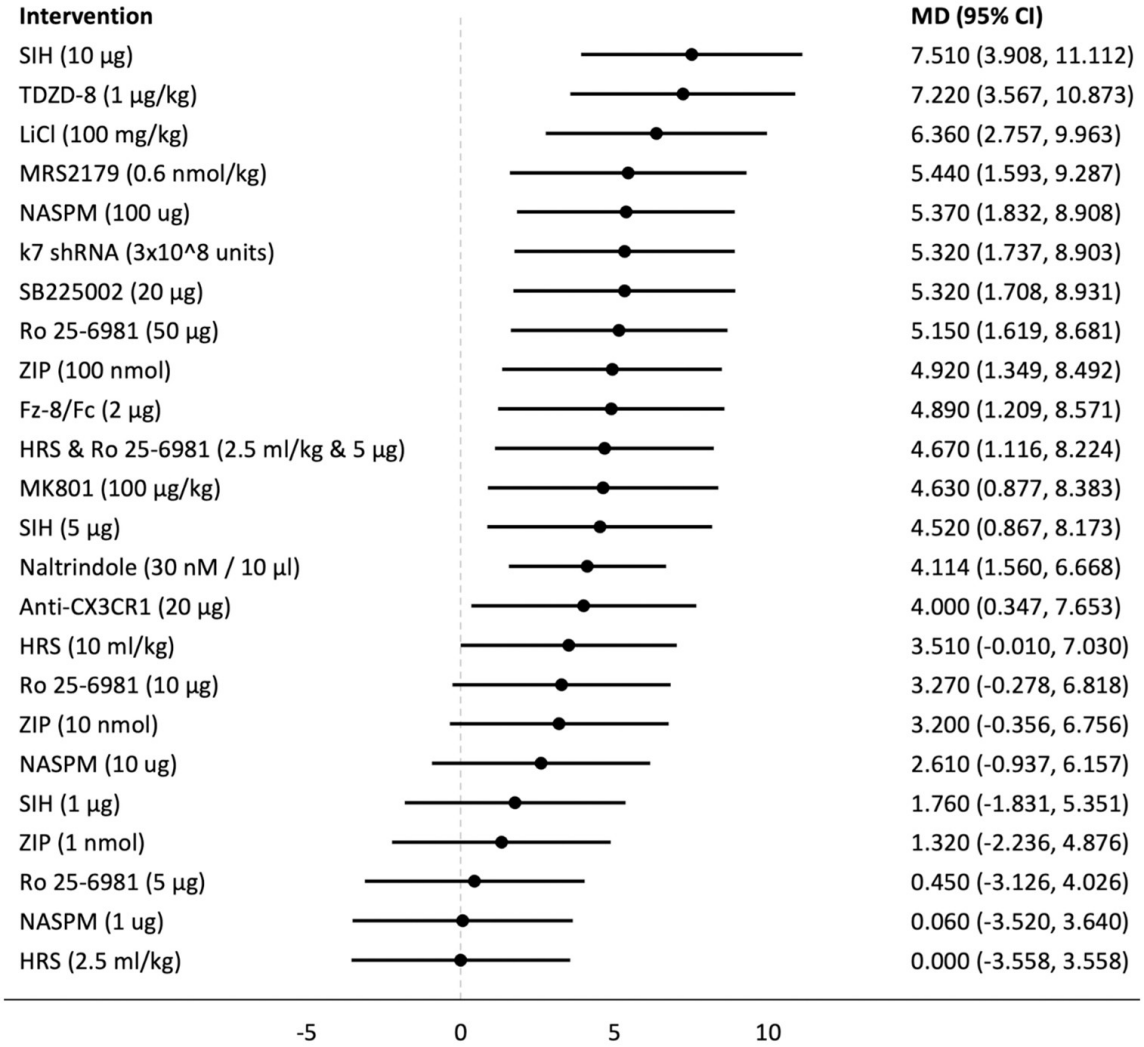
Forest plot for Group 2B. Approximately two thirds of the interventions provided a significant increase in PWL compared to the no-treatment condition. Most of the CIs in this subgroup are larger compared to the CIs of other subgroups. SIH (10 μg) had the largest mean difference.

**Fig 7:**
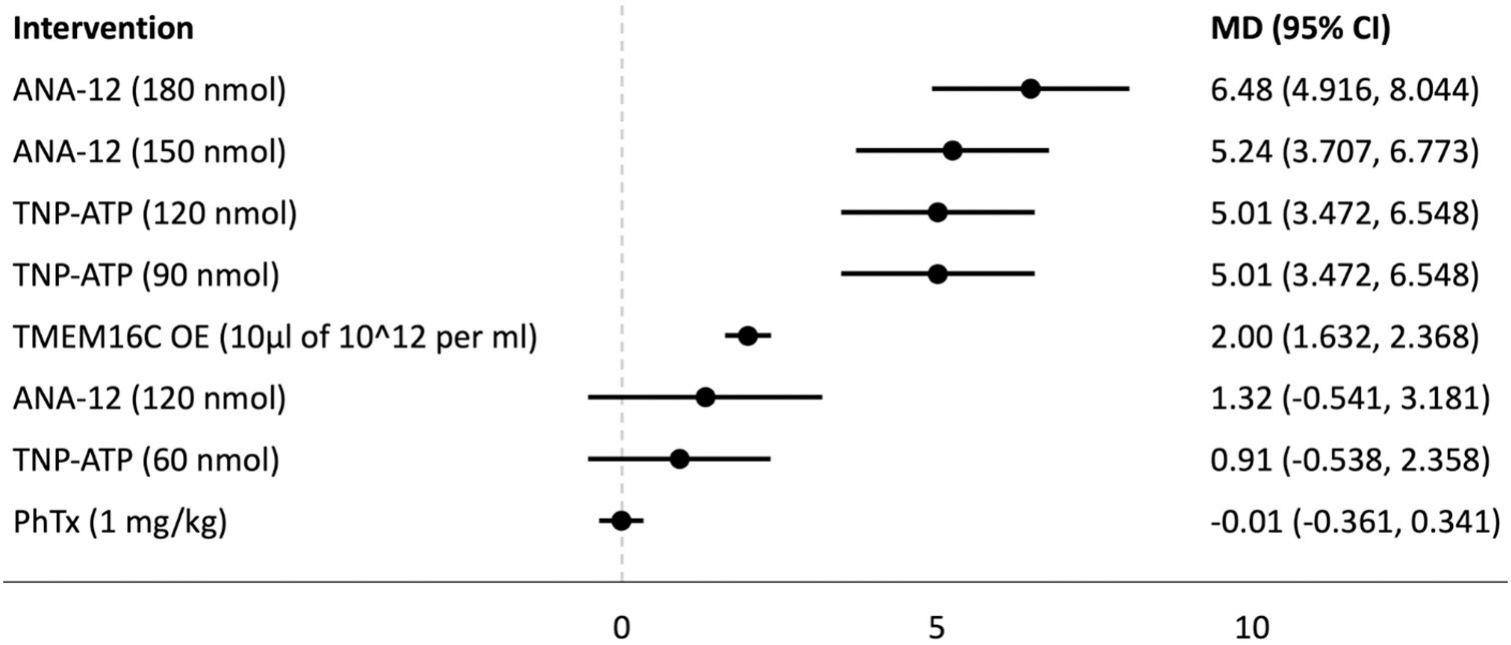
Forest plot for Group 2C. Approximately two thirds of the interventions provided a significant increase in PWL compared to the no-treatment condition. ANA-12 (180 nmol) had the largest mean difference.

**Fig 8:**
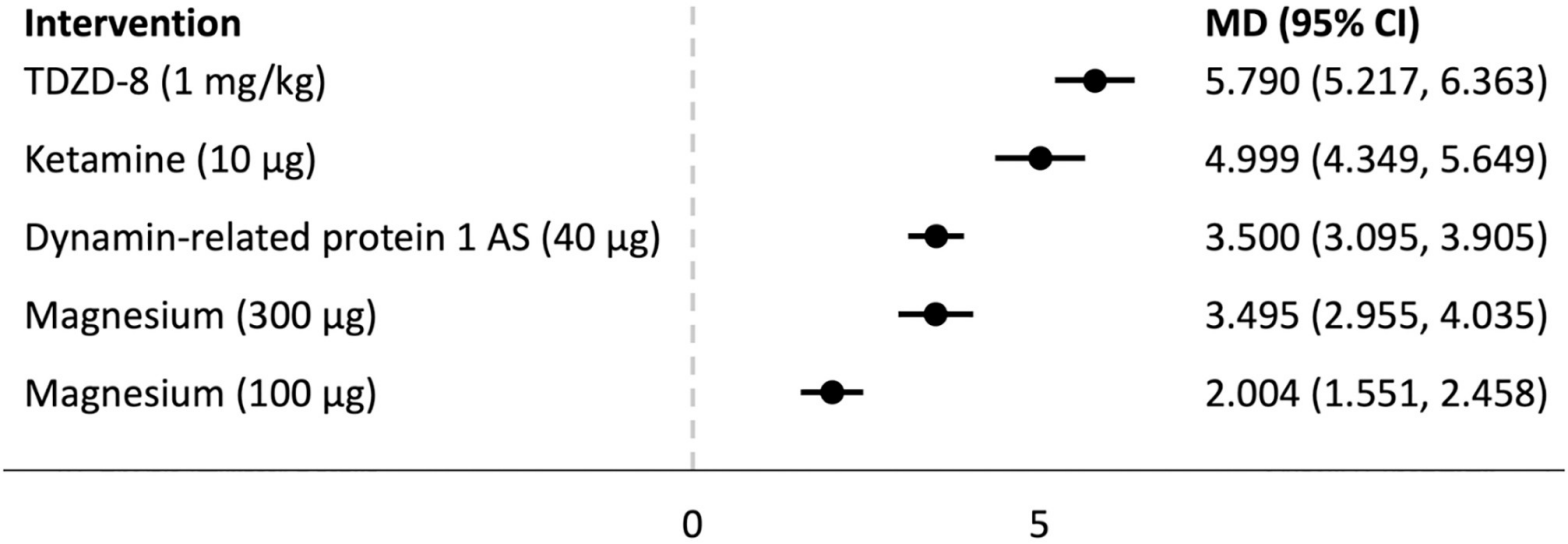
Forest plot for Group 3A. All of the interventions provided a significant increase in PWT compared to the no-treatment condition. TDZD-8 (1 mg/kg) had the largest mean difference.

**Fig 9:**
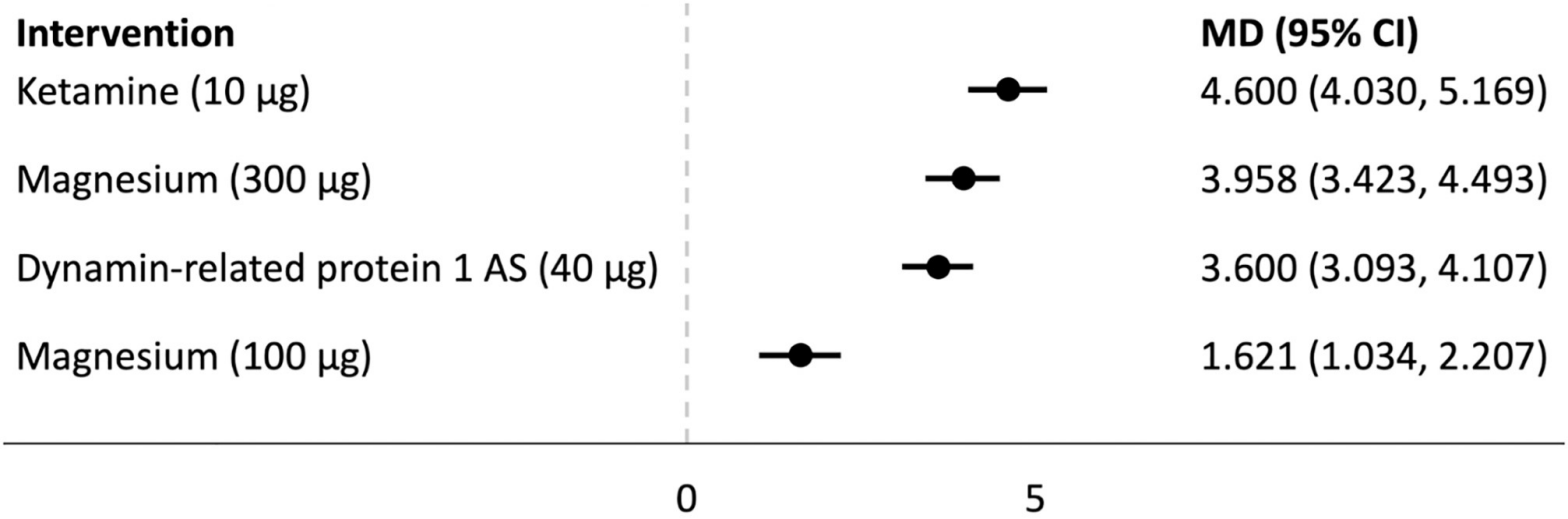
Forest plot for Group 3B. All of the interventions provided a significant increase in PWT compared to the no-treatment condition. Ketamine (10 μg) had the largest mean difference.

**Fig 10:**
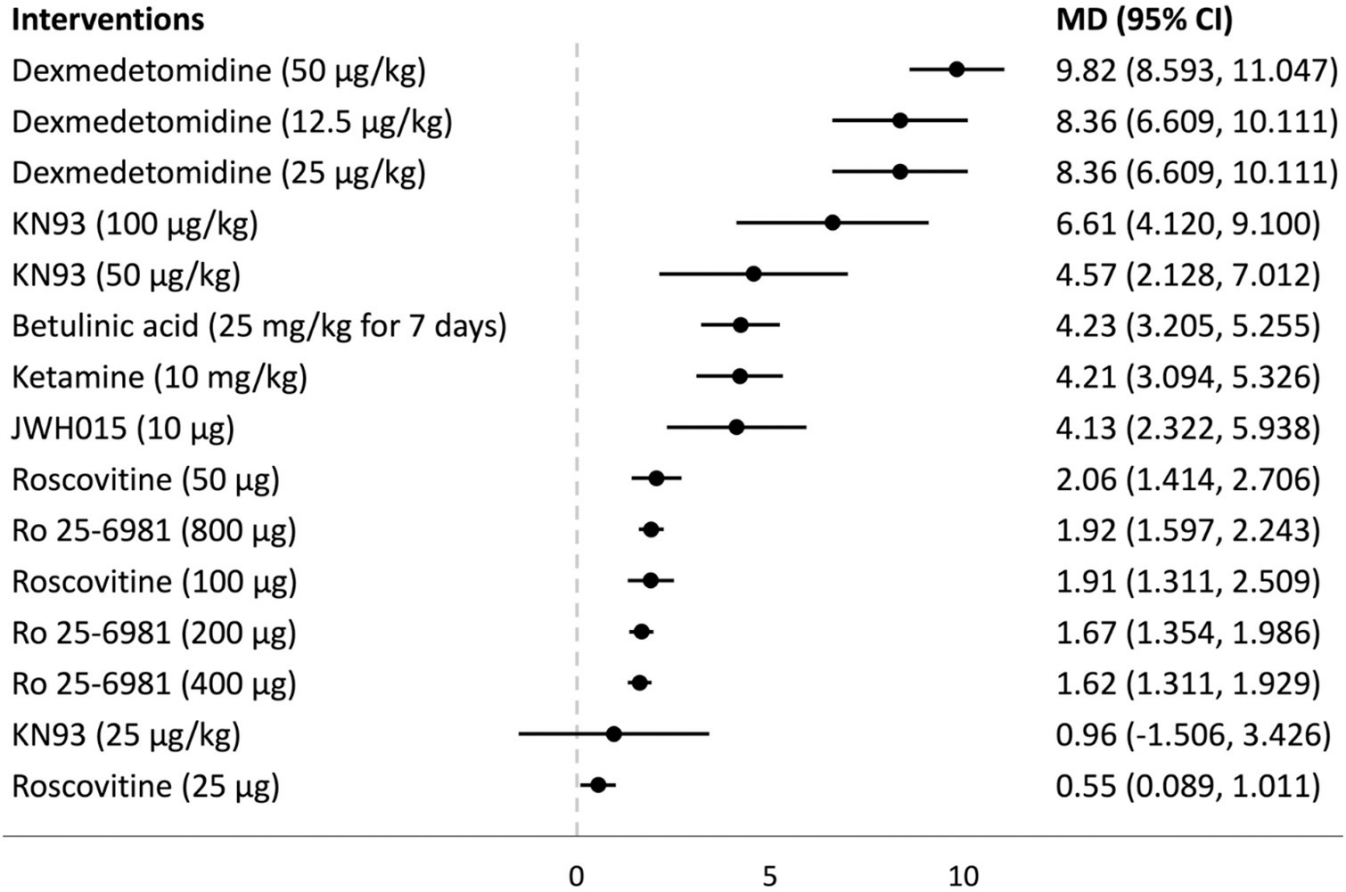
Forest plot for Group 4A. All of the interventions apart from KN93 (25 μg/kg) provided a significant increase in PWT compared to the no-treatment condition. Dexmedetomidine (50 μg/kg) had the largest mean difference.

**Fig 11:**
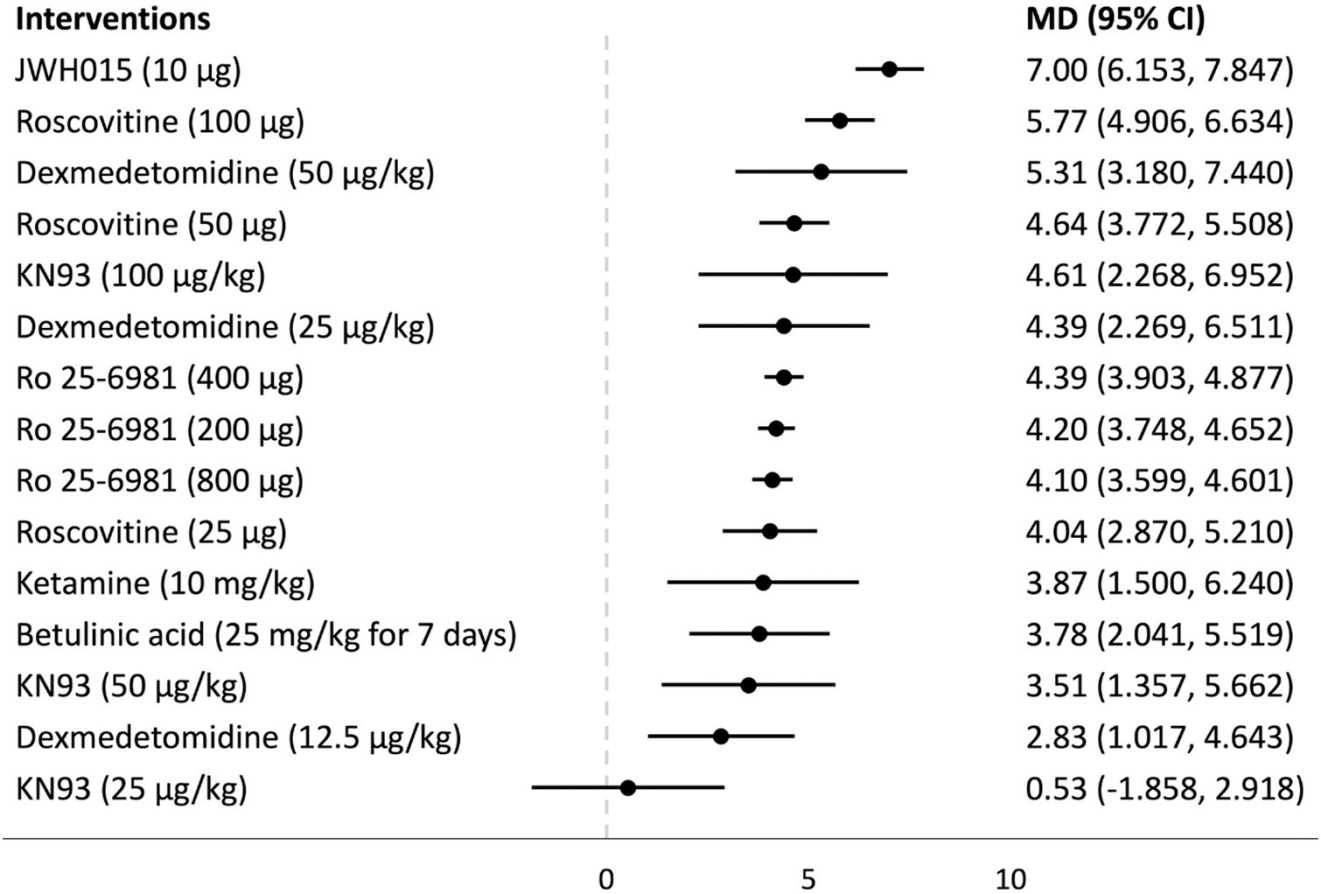
Forest plot for Group 4B. All of the interventions apart from KN93 (25 μg/kg) provided a significant increase in PWL compared to the no-treatment condition. JWH015 (10 μg) had the largest mean difference.

**Fig 12:**
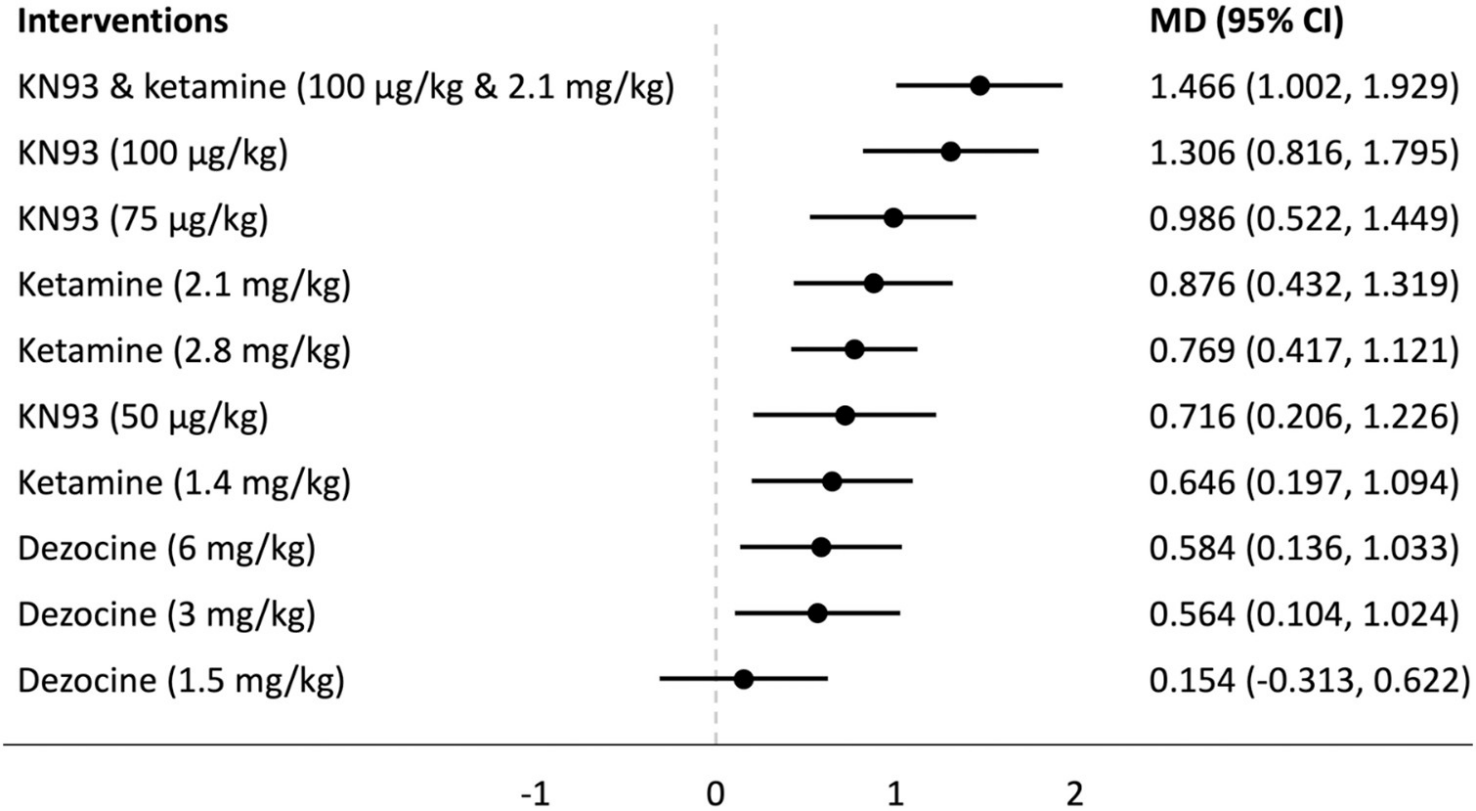
Forest plot for Group 5A. All of the interventions apart from dezocine (1.5 mg/kg) provided a significant increase in PWT compared to the no-treatment condition. KN93 & ketamine (100 μg/kg & 2.1 mg/kg) had the largest mean difference.

**Fig 13:**
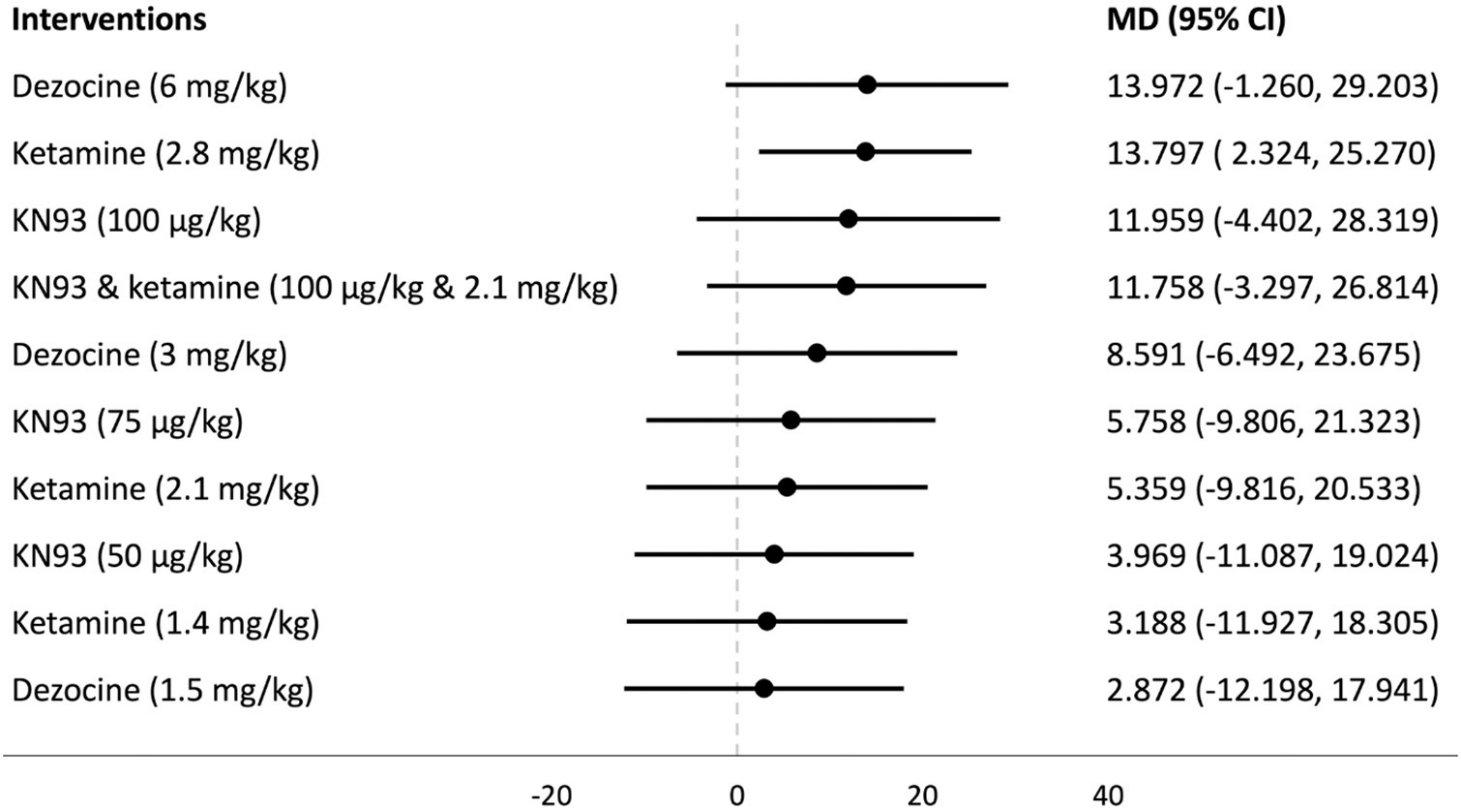
Forest plot for Group 5B. Dezocine (6 mg/kg) had the largest mean difference but its CI extends below 0. Ketamine (2.8 mg/kg) was the only intervention that provided a significant increase in PWL compared to the no-treatment condition.

Forest plots for the analysed groups are shown in Figs 3–13. The y-axes represent the no-treatment condition where remifentanil was administered (with or without placebo/vehicle depending on the study) but no pharmacological intervention was administered. The positive mean differences represent an increase in paw-withdrawal threshold (PWT) in mechanical QST groups and paw-withdrawal latency (PWL) in thermal QST groups. In all groups, most of the interventions provided a significant increase in PWT or PWL compared to the no-treatment condition. In addition, the order of interventions in the forest plots (based on the mean differences) is different between mechanical QST groups and thermal QST groups.

### Ranking of the interventions

Lists of P-scores for the networks are shown in S6 Table. The interventions with P-scores closest to 1 are the most effective, and the interventions with P-scores closest to 0 are the least effective. It should be noted that the order of the interventions ranked based on P-scores is different to the order of the interventions in forest plots that are solely based on the mean differences. In all groups, it can be seen that the ranking based on the P-scores is different between mechanical QST groups and thermal QST groups. A summary of the best interventions and their mechanism of action is provided in Table 3. Anxa1_2-26_ (500 μg) in Group 1 was the only intervention that was ranked as the most effective in both thermal and mechanical QST groups.

**Table 3:** The best intervention in each group.

Group	The best intervention	Mechanism of action
Group 1A	Anxa1_2-26_ (500 μg)	Annexin A1-derived peptide that decreases CXCL12/CXCR4 expression
Group 1B	Anxa1_2-26_ (500 μg)	Annexin A1-derived peptide that decreases CXCL12/CXCR4 expression
Group 2A	MRS2179 (0.6 nmol/kg)	P2Y purinoceptor 1 antagonist
Group 2B	SIH (10 μg)	Iron chelator
Group 2C	ANA-12 (180 nmol)	TrkB specific antagonist
Group 3A	TDZD-8 (1 mg/kg)	Selective GSK-3β inhibitor
Group 3B	Ketamine (10 μg)	NMDAR blocker
Group 4A	Dexmedetomidine (50 μg/kg)	α2-adrenergic agonist
Group 4B	JWH015 (10 μg)	CB2R agonist
Group 5A	KN93 & ketamine (100 μg/kg & 2.1 mg/kg)	CaMKII inhibitor & NMDAR blocker
Group 5B	Ketamine (2.8 mg/kg)	NMDAR blocker

CXCL12: C-X-C motif chemokine 12, CXCR4: C-X-C chemokine receptor type 4, TrkB: Tropomyosin receptor kinase B, GSK-3β: Glycogen synthase kinase-3 beta, CB2R: Cannabinoid receptor type 2, CaMKII: Calcium–calmodulin (CaM)-dependent protein kinase II, NMDAR: N-methyl-D-aspartate receptor.

## Discussion

We conducted a systematic review and NMA of preclinical trials investigating pharmacological interventions for RIH. Overall, the 62 eligible trials tested 86 individual interventions and 6 combination interventions. Only half of the studies (32/62) investigated more than one intervention and most interventions (46/62) were only investigated in one study. Thirty-five studies were included in a NMA as they shared similar characteristics (e.g. plantar incision status, remifentanil characteristics, animal model, and QST type) with at least one other study. The best-ranked interventions in each network were Anxa1_2-26_, MRS2179, SIH, ANA-12, TDZD-8, ketamine, dexmedetomidine, JWH015, and the combination of KN93 and ketamine. Future trials should consider comparing new interventions to these—especially to Anxa1_2-26_ and MRS217 as they were ranked highest in the largest networks of interventions.

Our review demonstrates that a wide variety of pharmacological interventions have been tested for RIH. Yet, the efficacy of almost half of these interventions could not be compared in a NMA due to the heterogeneity between the trials. Thus, future preclinical trials that solely focus on demonstrating that a pharmacological intervention has efficacy in preventing RIH are unlikely to advance this field much. In the planning of new trials, priority should be placed on comparing the efficacy of different interventions. Each trial should consider the choice of remifentanil characteristics (route of administration, infusion rate, and infusion duration) and QST measurement time points to enable consistent comparisons between trials. Similarly, PWT or PWL should be measured until RIH has resolved as otherwise the measurements give an incomplete view of the efficacy of the interventions which may distort the ranking. In addition, trials should consider providing their QST measurement data in supplementary material so that their results do not have to be excluded from NMAs due to data presentation anomalies (e.g. median and IQR instead of mean and SD).

In the current literature, some interventions have only been tested using one dose or a limited range of doses, which means that the efficacy of some of the interventions listed in our review has not been fully characterised. This should be kept in mind when comparing the ranking of the interventions and before ruling out interventions as potential candidates for preventing RIH.

Only two of the reviewed studies included female rodents [30,31]. The effect of sex on the development of OIH and its prevention is not fully understood but several studies suggest that significant sex differences may be observed depending on the genetic background of the test subjects and opioid doses used. For example, in CD-1 mice 1.6 mg/kg/d morphine infusion results in more pronounced hyperalgesia in females compared to males while no significant difference exists after 40 mg/kg/d morphine infusion [93]. In comparison, there were no differences in hyperalgesia in C57BL/6J male and female mice after 40 mg/kg/d or 1.6 mg/kg/d morphine infusions [94]. It has also been shown that NMDA receptor antagonist MK-801 and melanocortin-1 receptor (MC1R) antagonist MSG606 reverse morphine-induced hyperalgesia sex-dependently in male and female mice, respectively [94]. Thus, future trials should include both sexes when investigating OIH.

One of the main strengths in our work is that studies of each analysed group were very similar to each other and had low risk of bias which makes the rankings robust. On the other hand, the main limitation is that the efficacy of the interventions needs to be evaluated in humans before clinicians can be provided with recommendations. Similarly, preclinical trials do not provide information about potential side effects which is crucial in selecting the best intervention.

In conclusion, the current literature is too heterogeneous to produce a clear answer on which intervention is likely to be the most effective in preventing remifentanil-induced hyperalgesia. Despite the heterogeneity, our findings suggest that investigators conducting preclinical and clinical trials should consider comparing new interventions to Anxa12-26 and MRS217 as these were ranked highest in the last networks of interventions. Future research in this field should prioritise finding the most effective intervention over testing the efficacy of new options so that guidelines for preventing OIH could be produced.

## Supporting information

S1 Checklist PRISMA NMA checklist of items to include when reporting a systematic review involving a network meta-analysis. (DOCX)

S1 Table Detailed search strategy. (DOCX)

S2 Table Characteristics of individual studies. (XLSX)

S3 Table Risk of bias of individual studies. (XLSX)

S4 Table Studies with more than one intervention. (DOCX)

S5 Table Interventions that have been investigated in more than one study. (DOCX)

S6 Table Interventions ranked based on P-scores. (XLSX)

S1 Fig Network plot for Group 1. The network plot for Group 1A and Group 1B is the same. Forty-seven intervention options from 13 studies are presented. All interventions have been compared with the no-intervention condition but otherwise direct comparisons are limited.
(TIFF)

S2 Fig Network plot for Group 2A. Thirty-two intervention options from 11 studies are presented. All interventions have been compared with the no-intervention condition but otherwise direct comparisons are limited.
(TIFF)

S3 Fig Network plot for Group 2B. Twenty-four intervention options from nine studies are presented. All interventions have been compared with the no-intervention condition but otherwise direct comparisons are limited. S3 Fig is similar to S2 Fig but it is missing TNP-ATP, ANA-12, TMEM16C over expression, and philanthotoxin-7,4 (PHTx) which were measured using radiant heat tests and are shown in S4 Fig.
(TIFF)

S4 Fig Network plot for Group 2C. Philanthotoxin-7,4 (PHTx) and TMEM16C over expression were studied by Li et al. (2021)[82] while the rest of the interventions were studied by Fu et al. (2021)[30].
(TIFF)

S5 Fig Network plot for Group 3A. Five intervention options from four studies are presented. Sun et al. (2016)[66] investigated ketamine (10 μg), magnesium (100 μg), and magnesium (300 μg) and Sun et al. (2017)[78] investigated magnesium (100 μg) and magnesium (300 μg).
(TIFF)

S6 Fig Network plot for Group 3B. Four intervention options from three studies are presented. Fig 8 is similar to Fig 7 but it is missing TDZD-8 (1 mg/kg) investigated by Yuan et al. (2013) as it was evaluated using a hotplate.
(TIFF)

S7 Fig Network plot for Group 4. The network plot for Group 4A and Group 4B is the same. Fifteen intervention options from seven studies are presented. All interventions have been compared with the no-intervention condition but otherwise direct comparisons are limited.
(TIFF)

S8 Fig Network plot for Group 5. The network plot for Group 5A and Group B is the same. Ten intervention options from two studies are presented. Both included studies provide direct comparisons with other interventions, no-intervention or ketamine (2.8 mg/kg).
(TIFF)

S1 Data (XLSX)

## References

[1] Adams TJ, Aljohani DM, Forget P. Perioperative opioids: a narrative review contextualising new avenues to improve prescribing. Br J Anaesth. 2023;130: 709–718. doi: 10.1016/j.bja.2023.02.037 37059626

[2] Hughes LM, Irwin MG, Nestor CC. Alternatives to remifentanil for the analgesic component of total intravenous anaesthesia: a narrative review. Anaesthesia. 2023;78: 620–625. doi: 10.1111/anae.15952 36562193

[3] Bohringer C, Astorga C, Liu H. The Benefits of Opioid Free Anesthesia and the Precautions Necessary When Employing It. Transl Perioper Pain Med. 2020;7: 152–157. 31712783 PMC6844148

[4] Yi P, Pryzbylkowski P. Opioid Induced Hyperalgesia. Pain Med. 2015;16: S32–S36. doi: 10.1111/pme.12914 26461074

[5] Colvin LA, Fallon MT. Opioid-induced hyperalgesia: a clinical challenge. BJA Br J Anaesth. 2010;104: 125–127. doi: 10.1093/bja/aep392 20086062

[6] Roeckel L-A, Le Coz G-M, Gavériaux-Ruff C, Simonin F. Opioid-induced hyperalgesia: Cellular and molecular mechanisms. Neuroscience. 2016;338: 160–182. doi: 10.1016/j.neuroscience.2016.06.029 27346146

[7] Tompkins DA, Campbell CM. Opioid-Induced Hyperalgesia: Clinically Relevant or Extraneous Research Phenomenon? Curr Pain Headache Rep. 2011;15: 129–136. doi: 10.1007/s11916-010-0171-1 21225380 PMC3165032

[8] Angst MS, Clark JD. Opioid-induced Hyperalgesia: A Qualitative Systematic Review. Anesthesiology. 2006;104: 570–587. doi: 10.1097/00000542-200603000-00025 16508405

[9] Fishbain DA, Cole B, Lewis JE, Gao J, Rosomoff RS. Do Opioids Induce Hyperalgesia in Humans? An Evidence-Based Structured Review. Pain Med. 2009;10: 829–839. doi: 10.1111/j.1526-4637.2009.00653.x 19594845

[10] Dello Russo C, Di Franco V, Tabolacci E, Cappoli N, Navarra P, Sollazzi L, et al. Remifentanil-induced hyperalgesia in healthy volunteers: a systematic review and meta-analysis of randomized controlled trials. Pain. 2024;165: 972–982. doi: 10.1097/j.pain.0000000000003119 38047761 PMC11017745

[11] Fletcher D, Martinez V. Opioid-induced hyperalgesia in patients after surgery: a systematic review and a meta-analysis. BJA Br J Anaesth. 2014;112: 991–1004. doi: 10.1093/bja/aeu137 24829420

[12] Salengros J-C, Huybrechts I, Ducart A, Faraoni D, Marsala C, Barvais L, et al. Different Anesthetic Techniques Associated with Different Incidences of Chronic Post-thoracotomy Pain: Low-Dose Remifentanil Plus Presurgical Epidural Analgesia is Preferable to High-Dose Remifentanil with Postsurgical Epidural Analgesia. J Cardiothorac Vasc Anesth. 2010;24: 608–616. doi: 10.1053/j.jvca.2009.10.006 20005744

[13] Raffa RB, Pergolizzi JV. Opioid-Induced Hyperalgesia: Is It Clinically Relevant for the Treatment of Pain Patients? Pain Manag Nurs. 2013;14: e67–e83. doi: 10.1016/j.pmn.2011.04.002 23972873

[14] Gong X, Fan R, Zhu Q, Ye X, Chen Y, Zhang M. Exercise Reduces Morphine-Induced Hyperalgesia and Antinociceptive Tolerance. Cao D-Y, editor. BioMed Res Int. 2021;2021: 1–8. doi: 10.1155/2021/6667474 34616845 PMC8490029

[15] Laboureyras E, Boujema MB, Mauborgne A, Simmers J, Pohl M, Simonnet G. Fentanyl-induced hyperalgesia and analgesic tolerance in male rats: common underlying mechanisms and prevention by a polyamine deficient diet. Neuropsychopharmacology. 2022;47: 599–608. doi: 10.1038/s41386-021-01200-5 34621016 PMC8674360

[16] Zhao H-Y, Liu L-Y, Cai J, Cui Y-J, Xing G-G. Electroacupuncture Treatment Alleviates the Remifentanil-Induced Hyperalgesia by Regulating the Activities of the Ventral Posterior Lateral Nucleus of the Thalamus Neurons in Rats. Neural Plast. 2018;2018: 6109723. doi: 10.1155/2018/6109723 30534151 PMC6252233

[17] Koponen ME, Forget P. Pharmacological Interventions for Opioid-Induced Hyperalgesia: A Scoping Review of Preclinical Trials. J Clin Med. 2022;11: 7060. doi: 10.3390/jcm11237060 36498635 PMC9735807

[18] Xie W-J, Hong J-S, Feng C-F, Chen H-F, Li W, Li Y-C. Pharmacological interventions for preventing opioid-induced hyperalgesia in adults after opioid-based anesthesia: a systematic review and network meta-analysis. Front Pharmacol. 2023;14. Available: https://www.frontiersin.org/articles/10.3389/fphar.2023.1199794. doi: 10.3389/fphar.2023.1199794 37426819 PMC10324676

[19] Heinl C, Drdla-Schutting R, Xanthos DN, Sandkühler J. Distinct Mechanisms Underlying Pronociceptive Effects of Opioids. J Neurosci. 2011;31: 16748–16756. doi: 10.1523/JNEUROSCI.3491-11.2011 22090501 PMC6633307

[20] Cabañero D, Campillo A, Célérier E, Romero A, Puig MM. Pronociceptive Effects of Remifentanil in a Mouse Model of Postsurgical Pain: Effect of a Second Surgery. Anesthesiology. 2009;111: 1334–1345. doi: 10.1097/ALN.0b013e3181bfab61 19934880

[21] Stahmer SA, Shofer FS, Marino A, Shepherd S, Abbuhl S. Do Quantitative Changes in Pain Intensity Correlate with Pain Relief and Satisfaction? Acad Emerg Med. 1998;5: 851–857. doi: 10.1111/j.1553-2712.1998.tb02811.x 9754496

[22] Hutton B, Salanti G, Caldwell DM, Chaimani A, Schmid CH, Cameron C, et al. The PRISMA Extension Statement for Reporting of Systematic Reviews Incorporating Network Meta-analyses of Health Care Interventions: Checklist and Explanations. Ann Intern Med. 2015;162: 777–784. doi: 10.7326/M14-2385 26030634

[23] van der Mierden S, Hooijmans CR, Tillema AH, Rehn S, Bleich A, Leenaars CH. Laboratory animals search filter for different literature databases: PubMed, Embase, Web of Science and PsycINFO. Lab Anim. 2022;56: 279–286. doi: 10.1177/00236772211045485 34559023 PMC9194806

[24] Deuis JR, Dvorakova LS, Vetter I. Methods Used to Evaluate Pain Behaviors in Rodents. Front Mol Neurosci. 2017;10: 284. doi: 10.3389/fnmol.2017.00284 28932184 PMC5592204

[25] Cowie AM, Stucky CL. A Mouse Model of Postoperative Pain. Bio-Protoc. 2019;9: e3140. doi: 10.21769/BioProtoc.3140 30820443 PMC6390978

[26] Hooijmans CR, Rovers MM, de Vries RB, Leenaars M, Ritskes-Hoitinga M, Langendam MW. SYRCLE’s risk of bias tool for animal studies. BMC Med Res Methodol. 2014;14: 43. doi: 10.1186/1471-2288-14-43 24667063 PMC4230647

[27] Papakonstantinou T, Nikolakopoulou A, Higgins JPT, Egger M, Salanti G. CINeMA: Software for semiautomated assessment of the confidence in the results of network meta-analysis. Campbell Syst Rev. 2020;16: e1080. doi: 10.1002/cl2.1080 37131978 PMC8356302

[28] Rücker G, Schwarzer G. Ranking treatments in frequentist network meta-analysis works without resampling methods. BMC Med Res Methodol. 2015;15: 58. doi: 10.1186/s12874-015-0060-8 26227148 PMC4521472

[29] Metelli S, Chaiman A. NMAstudio: a fully interactive web-application for producing and visualising network meta-analyses. SRSM Annual Meeting 2021, Bern, Switzerland.; 2021. https://www.nmastudioapp.com/doc.

[30] Fu R, Li S, Li S, Gong X, Zhou G, Wang Y, et al. P2X4 receptor in the dorsal horn contributes to BDNF/TrkB and AMPA receptor activation in the pathogenesis of remifentanil-induced postoperative hyperalgesia in rats. Neurosci Lett. 2021;750: 135773. doi: 10.1016/j.neulet.2021.135773 33639220

[31] Mert T, Oksuz H, Tugtag B, Kilinc M, Senoglu N, Bilgin R. Modulating actions of NMDA receptors on pronociceptive effects of locally injected remifentanil in diabetic rats. Pharmacol Rep. 2014;66: 1065–1072. doi: 10.1016/j.pharep.2014.07.004 25443736

[32] Zhang L, Shu R, Wang H, Yu Y, Wang C, Yang M, et al. Hydrogen-rich saline prevents remifentanil-induced hyperalgesia and inhibits MnSOD nitration via regulation of NR2B-containing NMDA receptor in rats. Neuroscience. 2014;280: 171–180. doi: 10.1016/j.neuroscience.2014.09.024 25241067

[33] Zhang W, Liu Y, Hou B, Gu X, Ma Z. Activation of spinal alpha-7 nicotinic acetylcholine receptor attenuates remifentanil-induced postoperative hyperalgesia. Int J Clin Exp Med. 2015;8: 1871–1879. 25932115 PMC4402762

[34] Qi F, Liu T, Zhang X, Gao X, Li Z, Chen L, et al. Ketamine reduces remifentanil-induced postoperative hyperalgesia mediated by CaMKII-NMDAR in the primary somatosensory cerebral cortex region in mice. Neuropharmacology. 2020;162: 107783. doi: 10.1016/j.neuropharm.2019.107783 31541650

[35] Gao Y, Zhan W, Jin Y, Chen X, Cai J, Zhou X, et al. KCC2 receptor upregulation potentiates antinociceptive effect of GABAAR agonist on remifentanil-induced hyperalgesia. Mol Pain. 2022;18: 174480692210828. doi: 10.1177/17448069221082880 35352582 PMC8972932

[36] Zhang L, Zhao Y, Gao T, Zhang H, Li J, Wang G, et al. Artesunate Reduces Remifentanil-induced Hyperalgesia and Peroxiredoxin-3 Hyperacetylation via Modulating Spinal Metabotropic Glutamate Receptor 5 in Rats. Neuroscience. 2022;487: 88–98. doi: 10.1016/j.neuroscience.2022.01.003 35026318

[37] Yuan Y, Zhao Y, Shen M, Wang C, Dong B, Xie K, et al. Spinal NLRP3 inflammasome activation mediates IL-1β release and contributes to remifentanil-induced postoperative hyperalgesia by regulating NMDA receptor NR1 subunit phosphorylation and GLT-1 expression in rats. Mol Pain. 2022;18: 174480692210930. doi: 10.1177/17448069221093016 35322721 PMC9703502

[38] Wang Z, Yao Y, Tao Y, Fan P, Yu Y, Xie K, et al. Spinal microRNA-134-5p targets glutamate receptor ionotropic kainate 3 to modulate opioid induced hyperalgesia in mice. Mol Pain. 2023;19: 174480692311782. doi: 10.1177/17448069231178271 37247385 PMC10240872

[39] Li J, Wang Q, Gao Y, Ma W, Sun Z, Yu Y, et al. Blocking SphK/S1P/S1PR1 axis signaling pathway alleviates remifentanil-induced hyperalgesia in rats. Neurosci Lett. 2023;801: 137131. doi: 10.1016/j.neulet.2023.137131 36801239

[40] Li T, Wang H, Wang J, Chen Y, Yang C, Zhao M, et al. Annexin 1 inhibits remifentanil-induced hyperalgesia and NMDA receptor phosphorylation via regulating spinal CXCL12/CXCR4 in rats. Neurosci Res. 2019;144: 48–55. doi: 10.1016/j.neures.2018.07.007 30120960

[41] Aguado D, Abreu M, Benito J, García-Fernández J, Gómez De Segura IA. Amitriptyline, minocycline and maropitant reduce the sevoflurane minimum alveolar concentration and potentiate remifentanil but do not prevent acute opioid tolerance and hyperalgesia in the rat: A randomised laboratory study. Eur J Anaesthesiol. 2015;32: 248–254. doi: 10.1097/EJA.0000000000000098 24849503

[42] Lv C-C, Xia M-L, Shu S-J, Chen F, Jiang L-S. Attenuation of Remifentanil-Induced Hyperalgesia by Betulinic Acid Associates with Inhibiting Oxidative Stress and Inflammation in Spinal Dorsal Horn. Pharmacology. 2018;102: 300–306. doi: 10.1159/000493144 30253391

[43] Li S, Zeng J, Wan X, Yao Y, Wu Y, Zhao N, et al. Enhancement of spinal dorsal horn neuron N-methyl-D-aspartate receptor phosphorylation as the mechanism of remifentanil-induced hyperalgesia: Roles of protein kinase C and calcium/calmodulin-dependent protein kinase II. Mol Pain. 2017;13: 174480691772378. 10.1177/1744806917723789 PMC5549877 28714352

[44] Zhang L-L, Shu R-C, Li N, Wang Z-F, Wang C-Y, Wang H-Y, et al. Involvement of CCL1/CCR8 in Spinal Cord Dorsal Horn in Remifentanil-Induced Hyperalgesia in Rats. J Anesth Perioper Med. 2015;2: 53–60. doi: 10.24015/JAPM.2015.0009

[45] Li N, Zhang L, Shu R, Ding L, Wang Z, Wang H, et al. Involvement of CCL3/CCR5 Signaling in Dorsal Root Ganglion in Remifentanil-induced Hyperalgesia in Rats. Clin J Pain. 2016;32: 702–710. doi: 10.1097/AJP.0000000000000319 26550961

[46] Qiang Z, Yu W. Chemokine CCL7 regulates spinal phosphorylation of GluA1-containing AMPA receptor via interleukin-18 in remifentanil-induced hyperalgesia in rats. Neurosci Lett. 2019;711: 134440. doi: 10.1016/j.neulet.2019.134440 31430547

[47] Wang C, Li Q, Jia Z, Zhang H, Li Y, Zhao Q, et al. Spinal caspase-6 contributes to remifentanil-induced hyperalgesia via regulating CCL21/CXCR3 pathway in rats. Neurosci Lett. 2020;721: 134802. doi: 10.1016/j.neulet.2020.134802 32014515

[48] Gao Y, Zhou S, Pan Y, Gu L, He Y, Sun J. Wnt3a Inhibitor Attenuates Remifentanil-Induced Hyperalgesia via Downregulating Spinal NMDA Receptor in Rats. J Pain Res. 2020;Volume 13: 1049–1058. doi: 10.2147/JPR.S250663 32547170 PMC7245459

[49] Gong G, Hu L, Qin F, Yin L, Yi X, Yuan L, et al. Spinal WNT pathway contributes to remifentanil induced hyperalgesia through regulating fractalkine and CX3CR1 in rats. Neurosci Lett. 2016;633: 21–27. doi: 10.1016/j.neulet.2016.09.006 27616703

[50] Zhu M, Yuan ST, Yu WL, Jia LL, Sun Y. CXCL13 regulates the trafficking of GluN2B-containing NMDA receptor via IL-17 in the development of remifentanil-induced hyperalgesia in rats. Neurosci Lett. 2017;648: 26–33. doi: 10.1016/j.neulet.2017.03.044 28359934

[51] Xia WS, Peng YN, Tang LH, Jiang LS, Yu LN, Zhou XL, et al. Spinal ephrin B / EphB signalling contributed to remifentanil‐induced hyperalgesia via NMDA receptor. Eur J Pain. 2014;18: 1231–1239. doi: 10.1002/j.1532-2149.2014.00478.x 24737575 PMC4232047

[52] Peng Y, Zang T, Zhou L, Ni K, Zhou X. COX-2 contributed to the remifentanil-induced hyperalgesia related to ephrinB/EphB signaling. Neurol Res. 2019;41: 519–527. doi: 10.1080/01616412.2019.1580459 30759061

[53] Zhou J, Qi F, Hu Z, Zhang L, Li Z, Wang ZJ, et al. Dezocine attenuates the remifentanil-induced postoperative hyperalgesia by inhibition of phosphorylation of CaMKⅡα. Eur J Pharmacol. 2020;869: 172882. doi: 10.1016/j.ejphar.2019.172882 31863769

[54] Shu R, Zhang L, Zhang H, Li Y, Wang C, Su L, et al. NMDA Receptor Modulates Spinal Iron Accumulation Via Activating DMT1(-)IRE in Remifentanil-Induced Hyperalgesia. J Pain. 2021;22: 32–47. doi: 10.1016/j.jpain.2020.03.007 32574785

[55] Zheng Y, Cui S, Liu Y, Zhang J, Zhang W, Zhang J, et al. Dexmedetomidine prevents remifentanil-induced postoperative hyperalgesia and decreases spinal tyrosine phosphorylation of N-methyl-d-aspartate receptor 2B subunit. Brain Res Bull. 2012;87: 427–431. doi: 10.1016/j.brainresbull.2012.01.009 22301064

[56] Yuan Y, Sun Z, Chen Y, Zheng Y, Xie K, He Y, et al. Prevention of Remifentanil Induced Postoperative Hyperalgesia by Dexmedetomidine via Regulating the Trafficking and Function of Spinal NMDA Receptors as well as PKC and CaMKII Level In Vivo and In Vitro. Blum D, editor. PLOS ONE. 2017;12: e0171348. doi: 10.1371/journal.pone.0171348 28182698 PMC5300256

[57] Zhou S, Pan Y, Zhang Y, Gu L, Ma L, Xu Q, et al. Antisense oligodeoxynucleotides against dynamin-related protein 1 reduce remifentanil-induced hyperalgesia by modulating spinal N-methyl-D-aspartate receptor expression in rats. Korean J Pain. 2023;36: 316–327. doi: 10.3344/kjp.22398 37183652 PMC10322665

[58] Horii Y, Matsuda M, Takemura H, Ishikawa D, Sawa T, Amaya F. Spinal and Peripheral Mechanisms Individually Lead to the Development of Remifentanil-induced Hyperalgesia. Neuroscience. 2020;446: 28–42. doi: 10.1016/j.neuroscience.2020.08.014 32818602

[59] Wang Z, Tao Y, Song C, Liu P, Wang C, Li Y, et al. Spinal hevin mediates membrane trafficking of GluA1-containing AMPA receptors in remifentanil-induced postoperative hyperalgesia in mice. Neurosci Lett. 2020;722: 134855. doi: 10.1016/j.neulet.2020.134855 32088196

[60] Zhang L, Shu R, Wang C, Wang H, Li N, Wang G. Hydrogen-rich saline controls remifentanil-induced hypernociception and NMDA receptor NR1 subunit membrane trafficking through GSK-3β in the DRG in rats. Brain Res Bull. 2014;106: 47–55. doi: 10.1016/j.brainresbull.2014.05.005 24951883

[61] Shu R-C, Zhang L-L, Wang C-Y, Li N, Wang H-Y, Xie K-L, et al. Spinal Peroxynitrite Contributes to Remifentanil-induced Postoperative Hyperalgesia *via* Enhancement of Divalent Metal Transporter 1 without Iron-responsive Element–mediated Iron Accumulation in Rats. Anesthesiology. 2015;122: 908–920. doi: 10.1097/ALN.0000000000000562 25501899

[62] Sun Y, Zhang W, Liu Y, Liu X, Ma Z, Gu X. Intrathecal Injection of JWH015 Attenuates Remifentanil-Induced Postoperative Hyperalgesia by Inhibiting Activation of Spinal Glia in a Rat Model. Anesth Analg. 2014;118: 841–853. doi: 10.1213/ANE.0000000000000146 24651239

[63] Zhang L, Guo S, Zhao Q, Li Y, Song C, Wang C, et al. Spinal Protein Kinase Mζ Regulates α-Amino-3-hydroxy-5-methyl-4-isoxazolepropionic Acid Receptor Trafficking and Dendritic Spine Plasticity *via* Kalirin-7 in the Pathogenesis of Remifentanil-induced Postincisional Hyperalgesia in Rats. Anesthesiology. 2018;129: 173–186. doi: 10.1097/ALN.0000000000002190 29578864

[64] Abreu M, Aguado D, Benito J, García-Fernández J, Segura IAGD. Hyperalgesia and increased sevoflurane minimum alveolar concentration induced by opioids in the rat: A randomised experimental study. Eur J Anaesthesiol. 2015;32: 232–241. doi: 10.1097/EJA.0000000000000188 25485881

[65] Gu X, Wu X, Liu Y, Cui S, Ma Z. Tyrosine Phosphorylation of the N-Methyl-D-Aspartate Receptor 2B Subunit in Spinal Cord Contributes to Remifentanil-Induced Postoperative Hyperalgesia: the Preventive Effect of Ketamine. Mol Pain. 2009;5: 1744-8069-5–76. doi: 10.1186/1744-8069-5-76 20042082 PMC2809057

[66] Sun J, Lin H, Feng X, Dong J, Ansong E, Xu X. A comparison of intrathecal magnesium and ketamine in attenuating remifentanil-induced hyperalgesia in rats. BMC Anesthesiol. 2015;16: 74. doi: 10.1186/s12871-016-0235-9 27599837 PMC5013621

[67] Jiang M, Zhang W, Cheng C, Ma Z, Gu X. Intrathecal injection of KN93 attenuates paradoxical remifentanil-induced postoperative hyperalgesia by inhibiting spinal CaMKII phosphorylation in rats. Pharmacol Biochem Behav. 2015;134: 35–41. doi: 10.1016/j.pbb.2015.04.015 25937575

[68] Ye L, Xiao L, Yang Sy, Duan Jj, Chen Y, Cui Y, et al. Cathepsin S in the spinal microglia contributes to remifentanil-induced hyperalgesia in rats. Neuroscience. 2017;344: 265–275. doi: 10.1016/j.neuroscience.2016.12.030 28039043

[69] Li Y, Tang X, Wang C, Hu N, Xie K, Wang H, et al. Glycogen Synthase Kinase-3β Inhibition Prevents Remifentanil-Induced Postoperative Hyperalgesia via Regulating the Expression and Function of AMPA Receptors. Anesth Analg. 2014;119: 978–987. doi: 10.1213/ANE.0000000000000365 25126703

[70] Cui W, Wang S, Han R, Wang Q, Li J. CaMKII Phosphorylation in Primary Somatosensory Cortical Neurons is Involved in the Inhibition of Remifentanil-induced Hyperalgesia by Lidocaine in Male Sprague-Dawley Rats. J Neurosurg Anesthesiol. 2016;28: 44–50. doi: 10.1097/ANA.0000000000000177 25811361

[71] Wang S, Cui W, Zeng M, Ren Y, Han S, Li J. The increased release of amino acid neurotransmitters of the primary somatosensory cortical area in rats contributes to remifentanil-induced hyperalgesia and its inhibition by lidocaine. J Pain Res. 2018;Volume 11: 1521–1529. doi: 10.2147/JPR.S168008 30147356 PMC6097504

[72] Cui W, Li Y, Li S, Yang W, Jiang J, Han S, et al. Systemic Lidocaine Inhibits Remifentanil-induced Hyperalgesia via the Inhibition of cPKCgamma Membrane Translocation in Spinal Dorsal Horn of Rats. J Neurosurg Anesthesiol. 2009;21: 318–325. doi: 10.1097/ANA.0b013e3181abbde5 19955894

[73] Liu Y, Ni Y, Zhang W, Sun Y-E, Ma Z, Gu X. N-acetyl-cysteine attenuates remifentanil-induced postoperative hyperalgesia via inhibiting matrix metalloproteinase-9 in dorsal root ganglia. Oncotarget. 2017;8: 16988–17001. doi: 10.18632/oncotarget.15217 28199982 PMC5370016

[74] Aguado D, Abreu M, Benito J, Garcia-Fernandez J, Gómez de Segura IA. Effects of Naloxone on Opioid-induced Hyperalgesia and Tolerance to Remifentanil under Sevoflurane Anesthesia in Rats. Anesthesiology. 2013;118: 1160–1169. doi: 10.1097/ALN.0b013e3182887526 23407105

[75] Liu A, Wang X, Wang H, Lv G, Li Y, Li H. Δ-opioid receptor inhibition prevents remifentanil-induced post‑operative hyperalgesia via regulating GluR1 trafficking and AMPA receptor function. Exp Ther Med. 2017 [cited 16 Jul 2023]. doi: 10.3892/etm.2017.5652 29434817 PMC5776522

[76] Wang C, Li Y, Wang H, Xie K, Shu R, Zhang L, et al. Inhibition of DOR prevents remifentanil induced postoperative hyperalgesia through regulating the trafficking and function of spinal NMDA receptors in vivo and in vitro. Brain Res Bull. 2015;110: 30–39. doi: 10.1016/j.brainresbull.2014.12.001 25498394

[77] Zhao Q, Zhang L, Shu R, Wang C, Yu Y, Wang H, et al. Involvement of Spinal PKMζ Expression and Phosphorylation in Remifentanil-Induced Long-Term Hyperalgesia in Rats. Cell Mol Neurobiol. 2017;37: 643–653. doi: 10.1007/s10571-016-0401-0 27380044 PMC11482079

[78] Sun J, Lin H, He G, Lin W, Yang J. Magnesium sulphate attenuate remifentanil-induced postoperative hyperalgesia via regulating tyrosine phosphorylation of the NR2B subunit of the NMDA receptor in the spinal cord. BMC Anesthesiol. 2017;17: 30. doi: 10.1186/s12871-017-0325-3 28222697 PMC5320626

[79] Zhang L, Shu R, Wang C, Wang H, Li N, Wang G. Hydrogen-rich saline controls remifentanil-induced hypernociception and NMDA receptor NR1 subunit membrane trafficking through GSK-3β in the DRG in rats. Brain Res Bull. 2014;106: 47–55. doi: 10.1016/j.brainresbull.2014.05.005 24951883

[80] Su L, Bai X, Niu T, Zhuang X, Dong B, Wang G, et al. P2Y1 purinergic receptor inhibition attenuated remifentanil-induced postoperative hyperalgesia via decreasing NMDA receptor phosphorylation in dorsal root ganglion. Brain Res Bull. 2021;177: 352–362. doi: 10.1016/j.brainresbull.2021.10.006 34653560

[81] Ye L, Xiao L, Bai X, Yang S, Li Y, Chen Y, et al. Spinal mitochondrial-derived ROS contributes to remifentanil-induced postoperative hyperalgesia via modulating NMDA receptor in rats. Neurosci Lett. 2016;634: 79–86. doi: 10.1016/j.neulet.2016.09.016 27637388

[82] Li Y, Zhang L, Li J, Wang C, Chen Y, Yuan Y, et al. A Role for Transmembrane Protein 16C/Slack Impairment in Excitatory Nociceptive Synaptic Plasticity in the Pathogenesis of Remifentanil-induced Hyperalgesia in Rats. Neurosci Bull. 2021;37: 669–683. doi: 10.1007/s12264-021-00652-5 33779892 PMC8099973

[83] Zhang W, Liu Y, Hou B, Gu X, Ma Z. Activation of spinal alpha-7 nicotinic acetylcholine receptor attenuates remifentanil-induced postoperative hyperalgesia. PMC4402762 25932115

[84] Gu W, Zhang W, Lei Y, Cui Y, Chu S, Gu X, et al. Activation of spinal alpha-7 nicotinic acetylcholine receptor shortens the duration of remifentanil-induced postoperative hyperalgesia by upregulating KCC2 in the spinal dorsal horn in rats. Mol Pain. 2017;13: 174480691770476. doi: 10.1177/1744806917704769 28425312 PMC6997724

[85] Jiang M, Zhang W, Ma Z, Gu X. Antinociception and prevention of hyperalgesia by intrathecal administration of Ro 25–6981, a highly selective antagonist of the 2B subunit of N-methyl-d-aspartate receptor. Pharmacol Biochem Behav. 2013;112: 56–63. doi: 10.1016/j.pbb.2013.09.007 24076088

[86] Liu X, Liu Y, Zhang J, Zhang W, Sun Y-E, Gu X, et al. Intrathecal administration of roscovitine prevents remifentanil-induced postoperative hyperalgesia and decreases the phosphorylation of N-methyl-d-aspartate receptor and metabotropic glutamate receptor 5 in spinal cord. Brain Res Bull. 2014;106: 9–16. doi: 10.1016/j.brainresbull.2014.04.008 24769228

[87] Lu A, Lei H, Li L, Lai L, Liang W, Xu S. Role of mitochondrial Ca ^2+^ uniporter in remifentanil-induced postoperative allodynia. Eur J Neurosci. 2018;47: 305–313. doi: 10.1111/ejn.13842 29363836

[88] Deng L, Zhang L, Zhao H, Song F, Chen G, Zhu H. The role of p38MAPK activation in spinal dorsal horn in remifentanil-induced postoperative hyperalgesia in rats. Neurol Res. 2016;38: 929–936. doi: 10.1080/01616412.2016.1219078 27687719

[89] Yang L, Xu G, Wang Y. Up-regulation of CXCL1 and CXCR2 contributes to remifentanil-induced hypernociception via modulating spinal NMDA receptor expression and phosphorylation in rats. Neurosci Lett. 2016;626: 135–141. doi: 10.1016/j.neulet.2015.12.044 26724371

[90] Li Y, Wang H, Xie K, Wang C, Yang Z, Yu Y, et al. Inhibition of Glycogen Synthase Kinase-3β Prevents Remifentanil-Induced Hyperalgesia via Regulating the Expression and Function of Spinal N-Methyl-D-Aspartate Receptors In Vivo and Vitro. PLOS ONE. 2013;8: e77790. doi: 10.1371/journal.pone.0077790 24147079 PMC3797695

[91] Yuan Y, Wang J, Yuan F, Xie K, Yu Y, Wang G. Glycogen Synthase Kinase-3β Contributes to Remifentanil-Induced Postoperative Hyperalgesia via Regulating N-Methyl-D-Aspartate Receptor Trafficking. Anesth Analg. 2013;116: 473–481. doi: 10.1213/ANE.0b013e318274e3f1 23267003

[92] Ishida R, Nikai T, Hashimoto T, Tsumori T, Saito Y. Intravenous Infusion of Remifentanil Induces Transient Withdrawal Hyperalgesia Depending on Administration Duration in Rats. Anesth Analg. 2012;114: 224–229. doi: 10.1213/ANE.0b013e318237f678 22025495

[93] Juni A, Klein G, Kowalczyk B, Ragnauth A, Kest B. Sex differences in hyperalgesia during morphine infusion: Effect of gonadectomy and estrogen treatment. Neuropharmacology. 2008;54: 1264–1270. doi: 10.1016/j.neuropharm.2008.04.004 18457849

[94] Juni A, Cai M, Stankova M, Waxman AR, Arout C, Klein G, et al. Sex-specific Mediation of Opioid-induced Hyperalgesia by the Melanocortin-1 Receptor. Anesthesiology. 2010;112: 181–188. doi: 10.1097/ALN.0b013e3181c53849 19996949 PMC4642894

