# Supplementary material for: Pharmacological interventions for remifentanil-induced hyperalgesia: A systematic review and network meta-analysis of preclinical trials
Source: PLoS One. 2024 Dec 5;19(12):e0313749. doi: 10.1371/journal.pone.0313749 (PMC11620364; doi:10.1371/journal.pone.0313749)
Supplement: S1 Table — (DOCX) [file pone.0313749.s002.docx]

**Supplementary Table 1. Detailed search strategy.**

|  | **Embase (via Ovid)** |
| --- | --- |
| #1 | opioid induced hyperalgesia/ or opioid induced hyperalgesia.mp. or remifentanil induced hyperalgesia.mp. or opioid induced hypersensitivity.mp. or remifentanil induced hypersensitivity.mp. or opioid induced pain sensitivity.mp. or remifentanil induced pain sensitivity.mp. or opioid withdrawal induced hyperalgesia.mp. or remifentanil withdrawal induced hyperalgesia.mp. or opioid withdrawal induced pain sensitivity.mp. or opioid withdrawal induced pain sensitivity.mp. or remifentanil withdrawal induced pain sensitivity.mp. |
| #2 | Filter for preclinical studies developed by Mierden et al. (2021) |
| #3 | drug therapy.mp. or drug therapy/ or drug treatment.mp. or drug intervention.mp. or pharmacological intervention.mp. or pharmacological treatment.mp. or pharmacotherapy.mp. or pharmacological therapy.mp. or treat*.mp. or intervention.mp. or intervention study/ or prevent*.mp. or prevention/ or therapeutic strategy.mp. or therapeutic strategies.mp. |
|  | #1 AND #2 AND #3 |
|  | **PubMed** |
| #1 | "opioid induced hyperalgesia"[tw] or “remifentanil induced hyperalgesia”[tw] or "opioid induced hypersensitivity"[tw] or "remifentanil induced hypersensitivity"[tw] or “opioid induced pain sensitivity”[tw] or “remifentanil induced pain sensitivity”[tw] or "opioid withdrawal induced hyperalgesia"[tw] or “remifentanil withdrawal induced hyperalgesia"[tw] or "opioid withdrawal induced pain sensitivity"[tw] or "opioid withdrawal induced pain sensitivity"[tw] or "remifentanil withdrawal induced pain sensitivity"[tw] |
| #2 | Filter for preclinical studies developed by Mierden et al. (2021) |
| #3 | “drug therapy”[mesh] or “drug therapy”[tw] or “drug treatment”[tw] or “drug intervention”[tw] or “pharmacological intervention”[tw] or “pharmacological treatment”[tw] or “pharmacotherapy”[tw] or “pharmacological therapy”[tw] or “treat*”[tw] or “intervention”[tw] or “intervention study”[tw] or “prevent*”[tw] or “therapeutic strategy”[tw] or “therapeutic strategies”[tw] |
|  | #1 AND #2 AND #3 |
|  | **Web of Science (via Clarivate)** |
| #1 | TS=("opioid induced hyperalgesia" or “remifentanil induced hyperalgesia” or "opioid induced hypersensitivity" or "remifentanil induced hypersensitivity" or “opioid induced pain sensitivity” or “remifentanil induced pain sensitivity” or "opioid withdrawal induced hyperalgesia" or “remifentanil withdrawal induced hyperalgesia" or "opioid withdrawal induced pain sensitivity" or "opioid withdrawal induced pain sensitivity" or "remifentanil withdrawal induced pain sensitivity") |
| #2 | Filter for preclinical studies developed by Mierden et al. (2021) |
| #3 | TS=(“drug therapy” or “drug treatment” or “drug intervention” or “pharmacological intervention” or “pharmacological treatment” or “pharmacotherapy” or “pharmacological therapy”or “treat*” or “intervention” or “intervention study” or “prevent*” or “therapeutic strategy” or “therapeutic strategies”) |
|  | #1 AND #2 AND #3 |
|  | **Google Scholar (via Publish or Perish)** |
|  | “opioid induced hyperalgesia”\|“remifentanil induced hyperalgesia”\|“opioid induced hypersensitivity” “preclinical”\|”mouse”\|”mice”\|”rat”\|”animal”\|”rats” “drug therapy”\|“pharmacotherapy”\|“treatment”\|“intervention”\|“prevention” |
|  | Limit to 200 articles |
