# Supplementary material for: Pharmacological interventions for remifentanil-induced hyperalgesia: A systematic review and network meta-analysis of preclinical trials
Source: PLoS One. 2024 Dec 5;19(12):e0313749. doi: 10.1371/journal.pone.0313749 (PMC11620364; doi:10.1371/journal.pone.0313749)
Supplement: S4 Table — (DOCX) [file pone.0313749.s005.docx]

**S4 Table. Studies with more than one intervention.**

| Study | N of interventions | Interventions |
| --- | --- | --- |
| Li et al. (2023) | 11 | SK-1  LT1002  CYM-5442  CYM-5478  CAY10444  FTY720  TASP0277308  SEW2871  ac-YVAD-CMK  MCC950  PBN |
| Horii et al. (2020) | 4 | Naloxone  Methylnaltrexone  FR167653  HOE-140 |
| Yuan et al. (2022) | 4 | IL-1ra  (+)-Naloxone  A438079  Ac-YVAD-CMK |
| Zhang et al. (2014)a | 3 | Hydrogen-rich saline  Ro 25-6981  Hydrogen-rich saline + Ro 25-6981 |
| Zhang et al. (2014)b | 3 | Hydrogen rich saline  MK801  TDZD-8 |
| Aguado et al. (2015) | 3 | Amitriptyline  Minocycline  Maropitant |
| Zhang et al. (2015)b | 3 | PHA-543613  PNU-120596  PHA-543613 + PNU-120596 |
| Li et al. (2017) | 3 | KN93  Chelerythrine  PD98059 |
| Ye et al. (2017) | 3 | LHVS  Minocycline  PBN |
| Zhao et al. (2017) | 3 | ZIP  NPC-15437  Ro25-6981 |
| Zhang et al. (2018) | 3 | ZIP  Kalirin-7 shRNA  NASPM |
| Peng et al. (2019) | 3 | EphB2-Fc  SC58125  NS398 |
| Qi et al. (2020) | 3 | Ketamine  KN93  Ketamine + KN93 |
| Wang et al. (2020)a | 3 | VEID-fmk  Anti-CCL21  NBI-74330 |
| Li et al. (2021) | 3 | TMEM16C overexpression  Philanthotoxin-7,4 |
| Gao et al. (2022) | 3 | Muscimol  CLP257  Muscimol + CLP257 |
| Zhang et al. (2022) | 3 | Artesunate  MPEP  Artesunate + MPEP |
| Li et al. (2014) | 2 | LiCl  TDZD-8 |
| Mert et al. (2014) | 2 | NMDA  MK801 |
| Xia et al. (2014) | 2 | EphB1-Fc  MK-801 |
| Gu et al. (2017) | 2 | PNU-120596  TrkB/Fc |
| Gong et al. (2016) | 2 | Fz-8/Fc  Anti-CX3CR1 |
| Li et al. (2016) | 2 | Anti-CLL3  Maraviroc |
| Sun et al. (2016) | 2 | Magnesium  Ketamine |
| Zhu et al. (2017) | 2 | Anti-CXCL13  Anti-IL17 |
| Li et al. (2019) | 2 | Anxa1_2-26_  AMD3100 |
| Qiang and Yu (2019) | 2 | Anti-CLL7  IL-18BP |
| Gao et al. (2020) | 2 | IWP-2  Ro 25-6981 |
| Fu et al. (2021) | 2 | TNP-ATP  ANA-12 |
| Su et al. (2021) | 2 | MK801  MRS2179 |
| Wang et al. (2023) | 2 | AgomiR-134  ACET |
