# Supplementary material for: Pharmacological interventions for remifentanil-induced hyperalgesia: A systematic review and network meta-analysis of preclinical trials
Source: PLoS One. 2024 Dec 5;19(12):e0313749. doi: 10.1371/journal.pone.0313749 (PMC11620364; doi:10.1371/journal.pone.0313749)
Supplement: S5 Table — (DOCX) [file pone.0313749.s006.docx]

**S5 Table. Interventions that have been investigated in more than one study.**

| Intervention | Number of investigations | Studied by |
| --- | --- | --- |
| Ketamine | 4 | Abreu et al. (2015)  Gu et al. (2009)  Sun et al. (2016)  Qi et al. (2020) |
| MK-801 | 4 | Mert et al. (2014)  Zhang et al. (2014)b  Xia et al. (2014)  Su et al. (2021) |
| Ro25-6981 | 4 | Zhao et al. (2017)  Jiang et al. (2013)  Zhang et al. (2014)a  Gao et al. (2020) |
| TDZD-8 | 4 | Li et al. (2013)  Zhang et al. (2014)b  Yuan et al. (2013)  Li et al. (2014) |
| Hydrogen rich saline | 3 | Zhang et al. (2014)b  Zhang et al. (2014)a  Shu et al. (2015) |
| KN93 | 3 | Li et al. (2017)  Jiang et al. (2015)  Qi et al. (2020) |
| Lidocaine | 3 | Cui et al. (2015)  Wang et al. (2018)  Cui et al. (2009) |
| Naloxone / (+)-naloxone | 3 | Aguado et al. (2013)  Yuan et al. (2022)  Horii et al. (2020) |
| PBN | 3 | Ye et al. (2017)  Li et al. (2023)  Ye et al. (2016) |
| Naltrindole | 2 | Wang et al. (2015)  Liu et al. (2018) |
| Magnesium | 2 | Sun et al. (2016)  Sun et al. (2017) |
| Minocycline | 2 | Aguado et al. (2015)  Ye et al. (2017) |
| PNU-120596 | 2 | Zhang et al. (2015)b  Gu et al. (2017) |
| Ac-YVAD-CMK | 2 | Yuan et al. (2022)  Li et al. (2023) |
| Dexmedetomidine | 2 | Zheng et al. (2012)  Yuan et al. (2017) |
| Zeta inhibitory peptide | 2 | Zhao et al. (2017)  Zhang et al. (2018) |
