# Supplementary figures and images for: Pharmacological interventions for remifentanil-induced hyperalgesia: A systematic review and network meta-analysis of preclinical trials
Source: PLoS One. 2024 Dec 5;19(12):e0313749. doi: 10.1371/journal.pone.0313749 (PMC11620364; doi:10.1371/journal.pone.0313749)

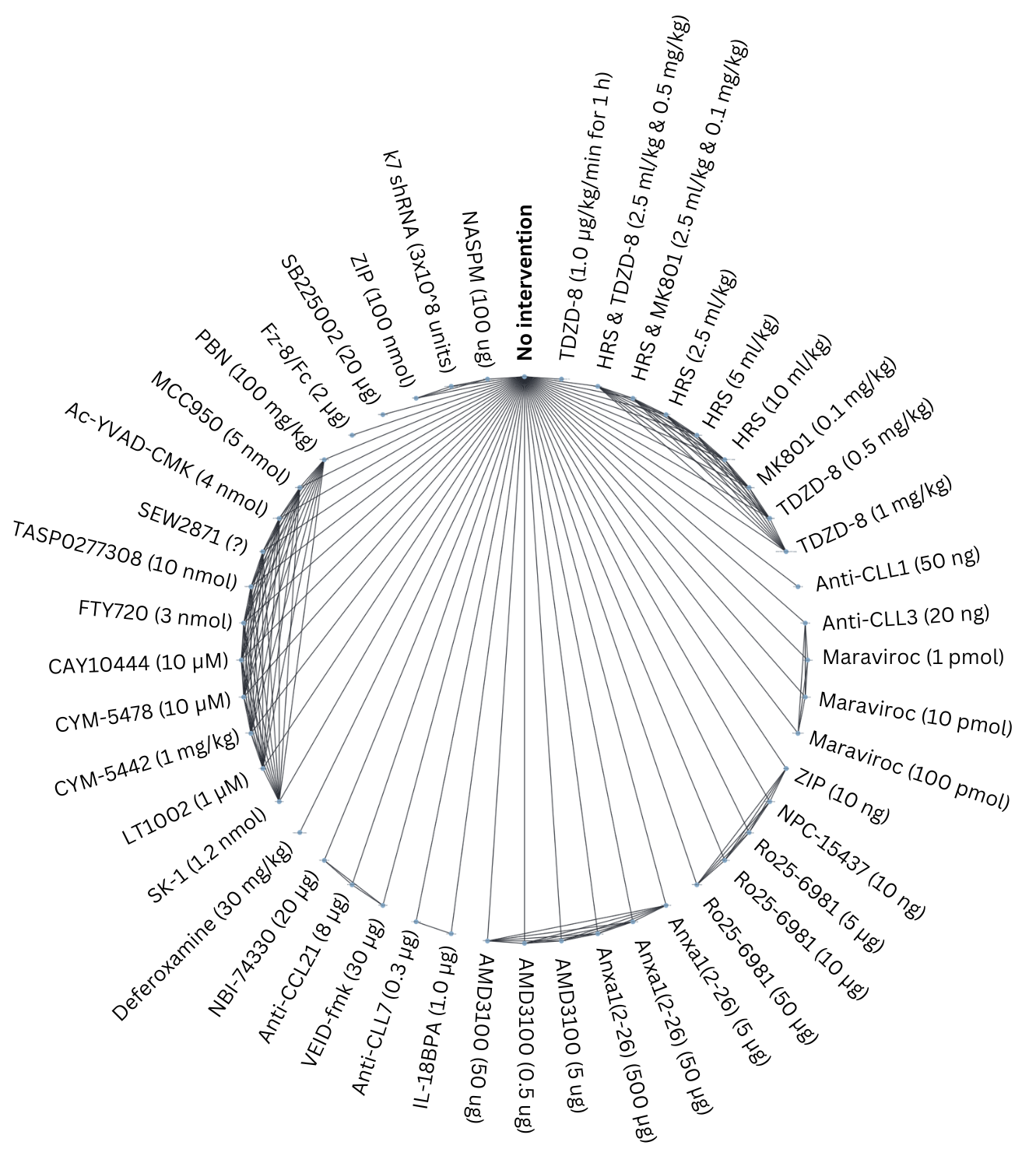

Supplement: S1 Fig — The network plot for Group 1A and Group 1B is the same. Forty-seven intervention options from 13 studies are presented. All interventions have been compared with the no-intervention condition but otherwise direct comparisons are limited. (TIFF) [file pone.0313749.s008.tiff]

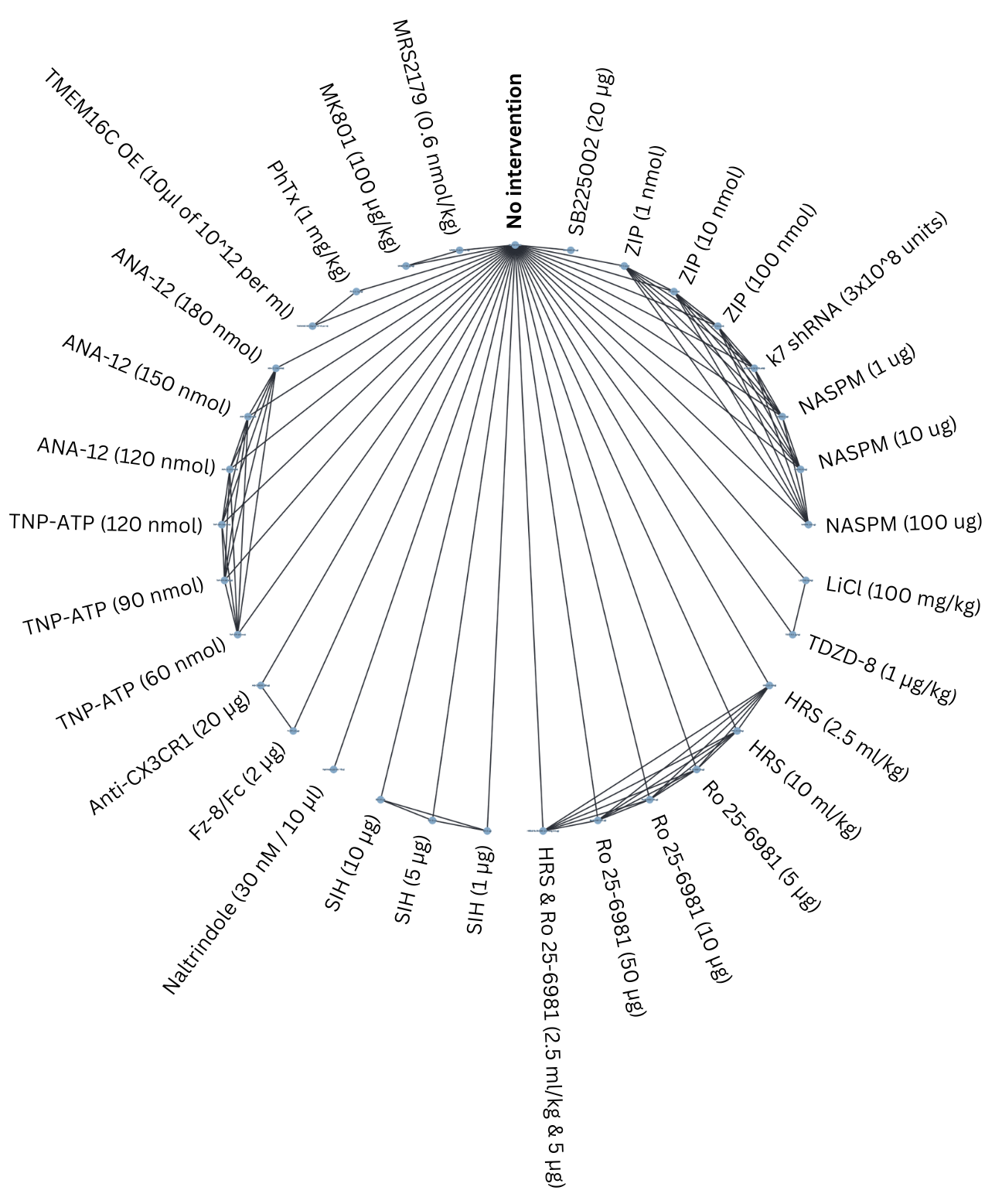

Supplement: S2 Fig — Thirty-two intervention options from 11 studies are presented. All interventions have been compared with the no-intervention condition but otherwise direct comparisons are limited. (TIFF) [file pone.0313749.s009.tiff]

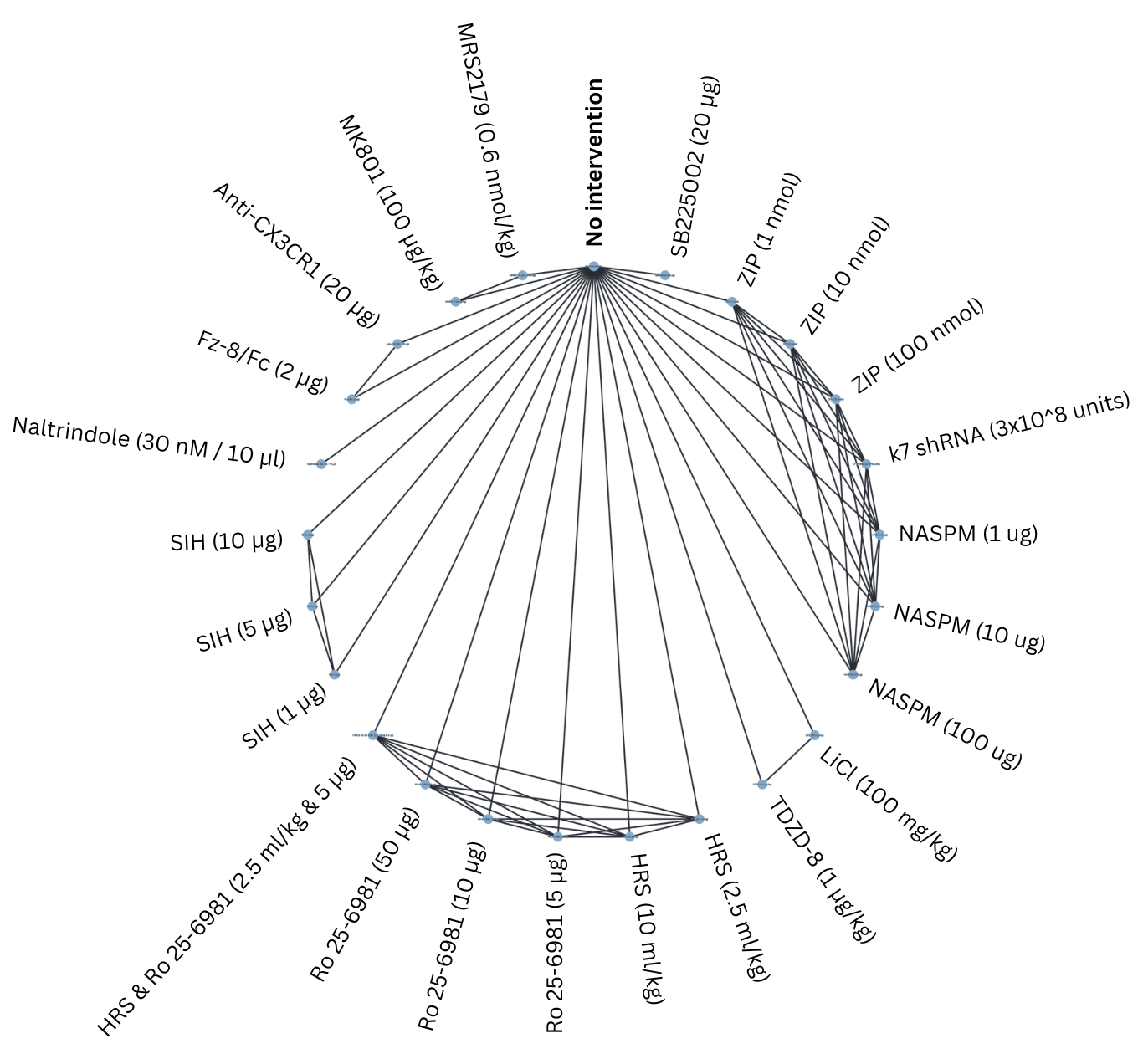

Supplement: S3 Fig — Twenty-four intervention options from nine studies are presented. All interventions have been compared with the no-intervention condition but otherwise direct comparisons are limited. S3 Fig is similar to S2 Fig but it is missing TNP-ATP, ANA-12, TMEM16C over expression, and philanthotoxin-7,4 (PHTx) which were measured using radiant heat tests and are shown in S4 Fig. (TIFF) [file pone.0313749.s010.tiff]

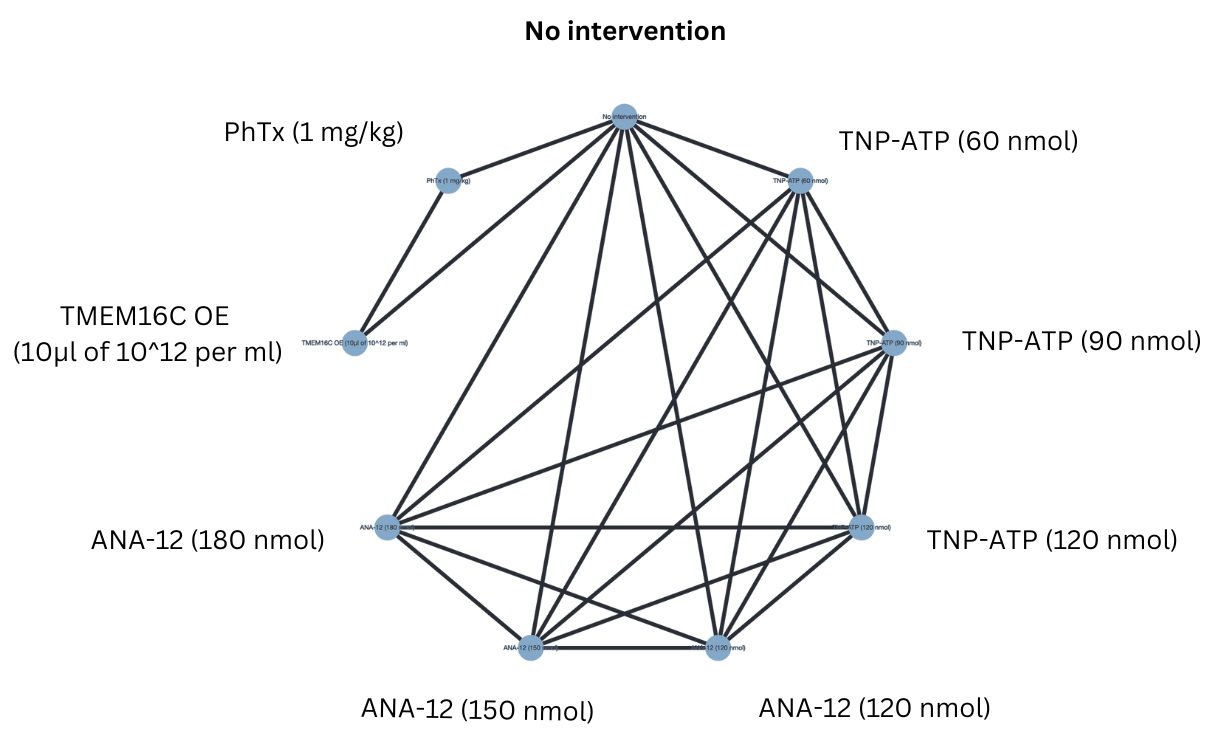

Supplement: S4 Fig — Philanthotoxin-7,4 (PHTx) and TMEM16C over expression were studied by Li et al. (2021)[82] while the rest of the interventions were studied by Fu et al. (2021)[30]. (TIFF) [file pone.0313749.s011.tiff]

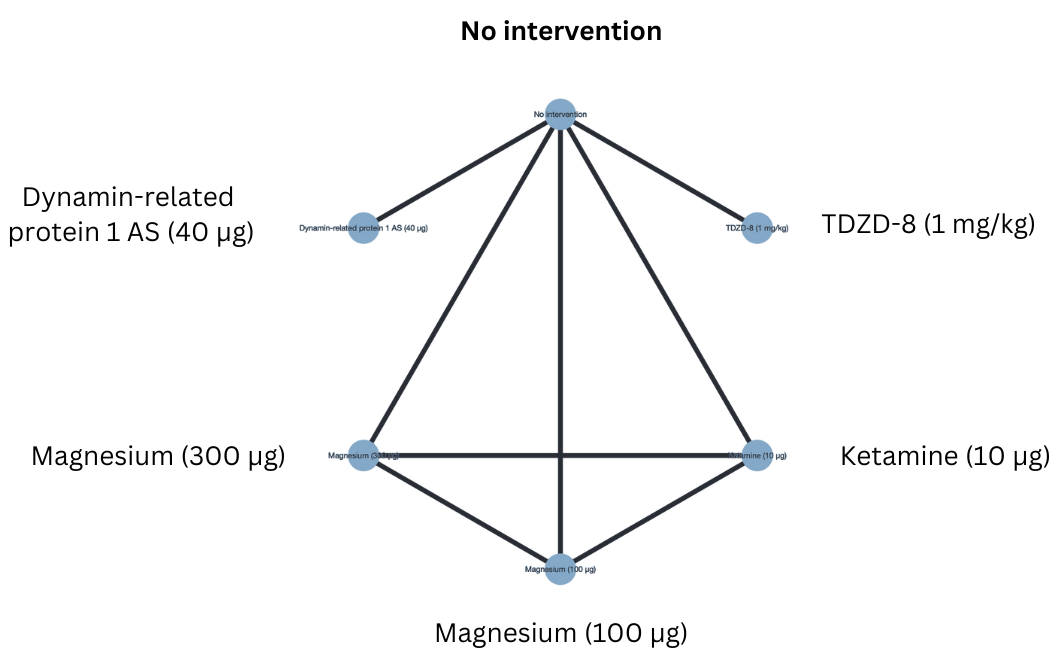

Supplement: S5 Fig — Five intervention options from four studies are presented. Sun et al. (2016)[66] investigated ketamine (10 μg), magnesium (100 μg), and magnesium (300 μg) and Sun et al. (2017)[78] investigated magnesium (100 μg) and magnesium (300 μg). (TIFF) [file pone.0313749.s012.tiff]

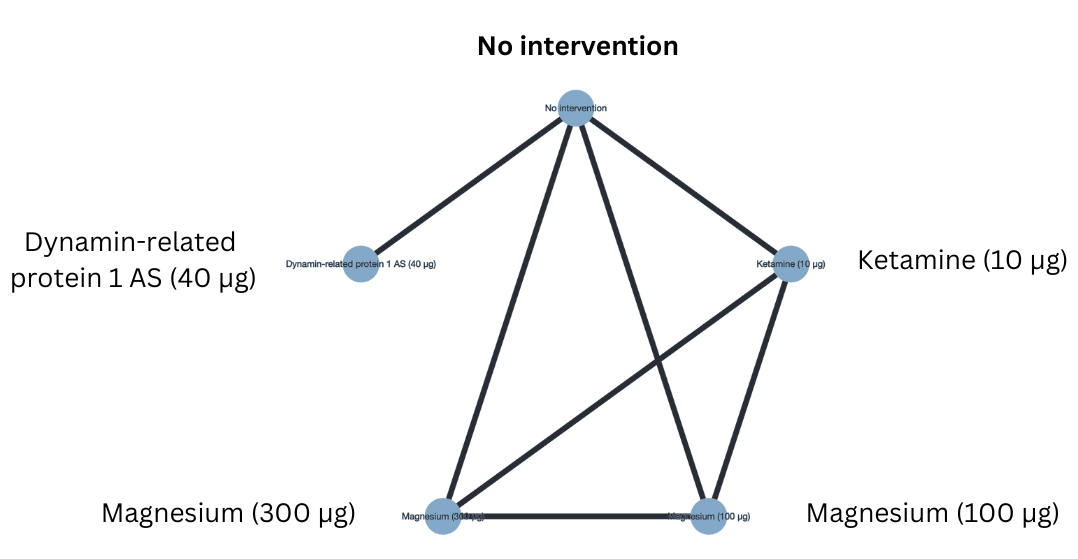

Supplement: S6 Fig — Four intervention options from three studies are presented. Fig 8 is similar to Fig 7 but it is missing TDZD-8 (1 mg/kg) investigated by Yuan et al. (2013) as it was evaluated using a hotplate. (TIFF) [file pone.0313749.s013.tiff]

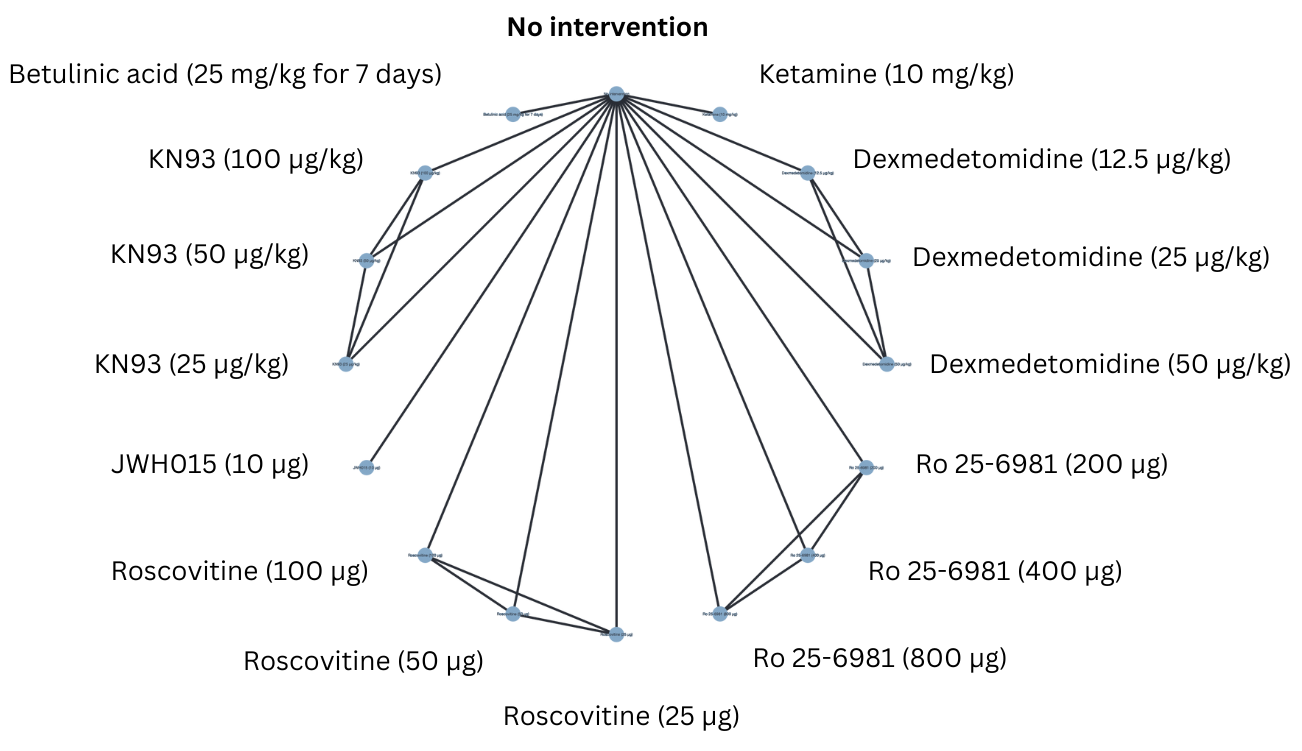

Supplement: S7 Fig — The network plot for Group 4A and Group 4B is the same. Fifteen intervention options from seven studies are presented. All interventions have been compared with the no-intervention condition but otherwise direct comparisons are limited. (TIFF) [file pone.0313749.s014.tiff]

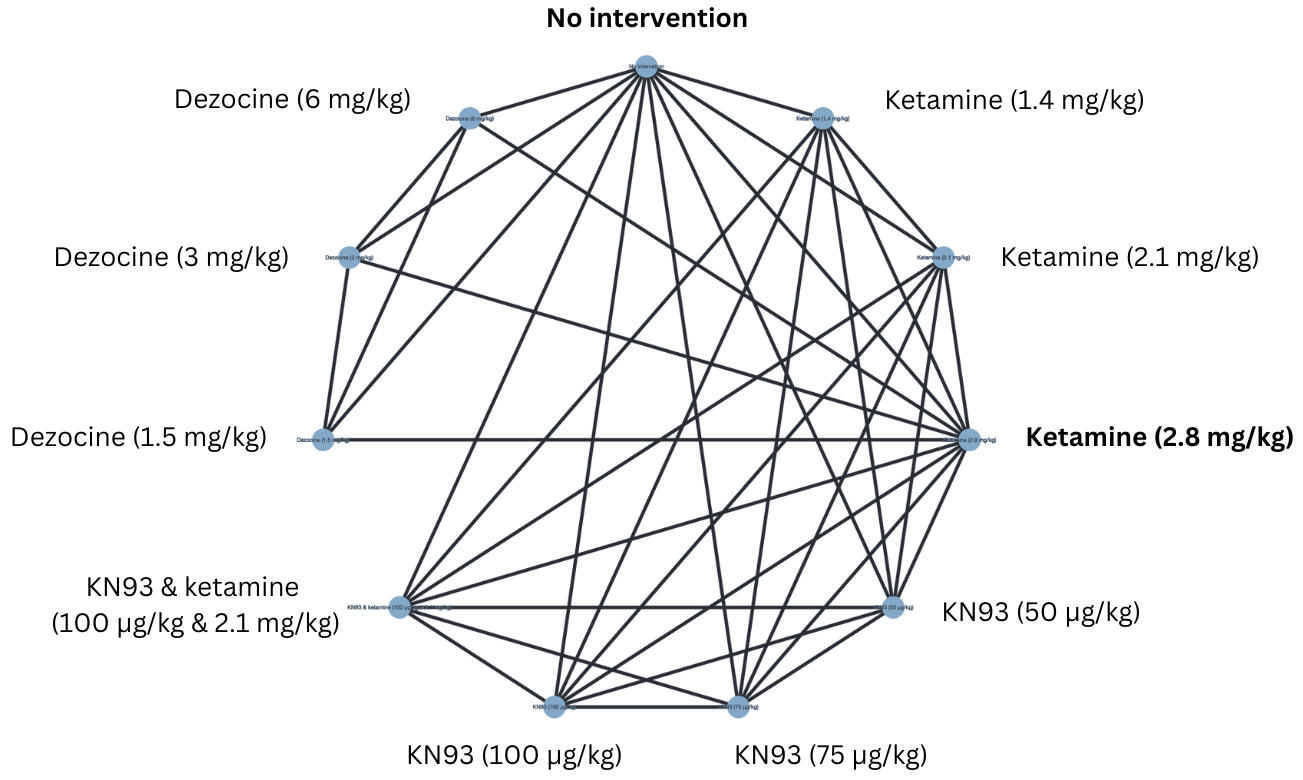

Supplement: S8 Fig — The network plot for Group 5A and Group B is the same. Ten intervention options from two studies are presented. Both included studies provide direct comparisons with other interventions, no-intervention or ketamine (2.8 mg/kg). (TIFF) [file pone.0313749.s015.tiff]
